# Uncovering Phenotypic Variation in Common Bean (*Phaseolus vulgaris* L.): Insights from the INCREASE Project

**DOI:** 10.3390/plants15081249

**Published:** 2026-04-18

**Authors:** Hourieh Tavakoli Hasanaklou, Lovro Sinkovič, Roberto Papa, Elena Bitocchi, Elisa Bellucci, Peter Dolničar, Barbara Pipan

**Affiliations:** 1 Crop Science Department, Agricultural Institute of Slovenia, Hacquetova ulica 17, SI-1000 Ljubljana, Slovenia; hourieh.tavakolihasanaklou@kis.si (H.T.H.); lovro.sinkovic@kis.si (L.S.); peter.dolnicar@kis.si (P.D.); 2 Department of Agricultural, Food and Environmental Sciences, Polytechnic University of Marche, via Brecce Bianche, 60131 Ancona, Italy; r.papa@staff.univpm.it (R.P.); e.bitocchi@univpm.it (E.B.); e.bellucci@univpm.it (E.B.)

**Keywords:** *Phaseolus vulgaris*, phenotypic diversity, germplasm evaluation, multivariate analysis, selection index, year-to-year variation, yield stability

## Abstract

The common bean (*Phaseolus vulgaris* L.) is a major food legume and an important plant genetic resource for sustainable agriculture. Effective use of this diversity requires integrated evaluation of phenotypic variation and agronomic performance, with preliminary assessments of line performance across seasons. In this study, phenotypic diversity was evaluated in a subsample of the INCREASE R-core collection, a large and well-defined core set of common-bean SSD lines derived from heterogeneous germplasm lines. A total of 507 lines were characterized using 57 agro-morphological traits. Multivariate analyses revealed wide phenotypic diversity structured mainly by growth habit, phenology, and yield-related traits, with clear differentiation among lines. Mixed-data clustering identified cluster 4 as the main phenotypic group associated with higher seed- and yield-related performance and composed predominantly of indeterminate climbing landraces. Multi-trait selection indices generally ranked lines from this group highest, while early, small-seeded types tended to show lower overall performance. Evaluation of a selected subset of 19 lines across two growing seasons revealed marked year-to-year variation in yield performance, indicating contrasting responses among otherwise high-performing lines. The multi-trait genotype–ideotype distance index further distinguished lines with balanced performance across traits and years. Overall, this study shows that large-scale phenotypic characterization combined with multi-trait evaluation can provide a useful exploratory basis for identifying breeding-relevant ideotypes and promising lines for further validation for common-bean improvement.

## 1. Introduction

The common bean (*Phaseolus vulgaris* L.) is one of the most important food legumes worldwide. It is a major source of protein, dietary fiber, and essential micronutrients for human nutrition [1,2,3]. Dry beans are the most widely consumed grain legume, mainly due to their high nutritional value, relatively high seed protein content, and long storage life [1,2]. In addition, the common bean grows well in marginal and low-input environments and improves soil fertility through biological nitrogen fixation, making it important for food security in resource-limited regions [2,4]. As such, the common bean is a key plant genetic resource supporting sustainable agriculture and agrobiodiversity conservation. The common bean was independently domesticated in Mesoamerica and the Andean region, resulting in two main gene pools with distinct genetic backgrounds and evolutionary histories [2,5,6]. These gene pools differ in key traits such as seed size and shape, growth habit (bush or climbing), flowering behavior, and adaptation to environmental stress as a result of domestication and breeding [2,7]. Studies based on morphological traits and genetic markers have revealed high diversity within the common bean across landraces and breeding lines, highlighting valuable sources of traits related to yield, growth habit, and local adaptation [8,9]. To better exploit this variability, recent studies have increasingly applied multivariate and multi-environment approaches for germplasm evaluation. Multivariate methods, such as Principal Component Analysis (PCA) and Factor Analysis of Mixed Data (FAMD), are widely used to describe phenotypic variation, group lines with similar traits, and support parental selection in breeding programs [8,10,11,12,13]. In parallel, multi-environment trials combined with statistical models have improved the evaluation of yield stability and genotype × environment interaction. Models such as the Additive Main Effects and Multiplicative Interaction (AMMI) model and the Genotype plus Genotype × Environment (GGE) biplot are commonly applied to identify genotypes with stable performance across environments [14,15,16]. These approaches enable a more comprehensive evaluation of common-bean ideotypes than traditional single trait or single-location studies [17].

Despite these methodological advances, selecting superior lines from large and diverse germplasm collections remains challenging, particularly when many qualitative and quantitative traits must be considered simultaneously. To support selection in such complex datasets, selection index (SI) approaches are widely used, as they allow multiple traits to be combined into a single value reflecting overall performance and breeding relevance [18,19]. These indices are commonly applied to rank lines in large populations. Both quantitative and qualitative traits, including agronomic performance, stress responses, and yield-related components, are considered in the selection process [8,20,21]. More recently, Olivoto and Nardino [22] proposed the multi-trait genotype–ideotype distance index (MGIDI) as a complementary selection tool designed to capture complex ideotype structures. The MGIDI integrates correlated traits into a single metric that represents the distance of each genotype from an ideal ideotype while accounting for trait correlations and trade-offs [11,22]. This approach has proven effective for identifying genotypes with balanced performance across yield, quality, and stress-related traits, particularly in smaller and well-defined datasets evaluated across environments [23]. Nevertheless, integrated studies remain limited in regard to the common bean. Few have jointly assessed phenotypic diversity, multi-trait performance, and yield stability across environments using large and structured germplasm panels. The INCREASE project’s R-core collection represents a large, well-defined, and diverse core set of common-bean lines [24]. It was developed within a standardized and coordinated framework, providing an opportunity to address this gap [25,26,27,28]. In this study, we combine extensive agro-morphological phenotyping with two-year yield evaluation under contrasting seasonal conditions. This approach allows us to assess phenotypic diversity, trait relationships, and performance stability within a subsample of the R-core collection. The objectives of the present study were to (1) characterize phenotypic diversity using a comprehensive set of quantitative and qualitative traits; (2) identify trait combinations and ideotypes associated with high yield and favorable phenotypic performance; and (3) examine year-to-year variation in yield and multi-trait performance using AMMI and GGE models together with the MGIDI index. This study provides new insights into common-bean diversity and offers an initial basis for breeding-oriented interpretation of yield stability and multi-trait performance.

## 2. Results

### 2.1. Composition of R-Core Collection

**Figure 1:**
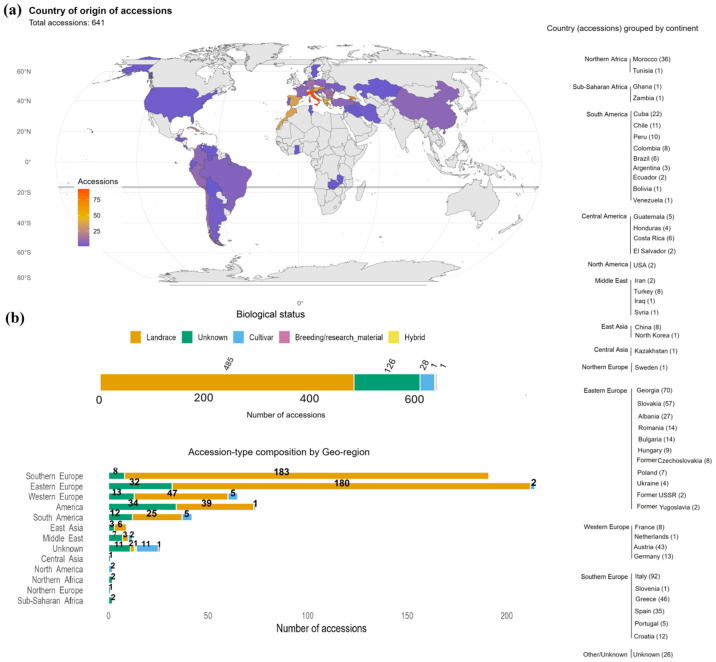
Geographic origin and biological status of the INCREASE R-core collection subsample. (**a**) Distribution of 641 lines across 13 geographic regions based on passport information. (**b**) Biological status categories (landrace, cultivar, hybrid, breeding/research material, unknown) for the same lines.

The subsample of the INCREASE R-core collection comprised 641 lines of *Phaseolus vulgaris*, derived by Single Seed Descent (SSD) from heterogeneous germplasm lines. Although the SSD procedure results in genetically fixed lines, the associated metadata refer to the original heterogeneous lines from which these lines were derived. Each SSD line was assigned a Digital Object Identifier (DOI) (Supplementary Table S6). This sample includes the 450 lines belonging to the INCREASE Training core (T-core), which is the nested collection of the R-core [24]. All lines were documented using standard passport descriptors, including geographic origin and biological status of the original lines. The collection had a predominantly European profile, with 471 lines (≈73.0%) derived from lines originating from Europe. Eastern Europe was the largest contributor (214 lines), followed by Southern Europe (191 lines). Western Europe contributed 65 lines, while Northern Europe was represented by a single line from Sweden. Outside Europe, 37 lines originated from North Africa, and 12.9% of the panel came from the Americas. Smaller numbers originated from Asia and Sub-Saharan Africa, and 4.1% of lines had no recorded origin (Figure 1a). The distribution of biological status showed a similar pattern (Figure 1b). Landraces were the dominant group, with 485 lines (≈75.7%). Cultivars (28), hybrids (1), and breeding or research materials (1) were rare. Another 126 lines (≈19.7%) lacked biological status information. Of the 641 planted lines, 507 reached flowering and produced complete phenotypic data (Supplementary Table S6A). The remaining 134 lines (20.9%) did not germinate or failed to reach reproductive growth and were excluded from phenotypic analyses but remain documented in the dataset (Supplementary Table S6B). The excluded lines could not be meaningfully included in trait-based analyses because most reproductive and yield-related descriptors were structurally missing for these lines.

### 2.2. Phenotypic Variation Across Quantitative Traits

The 20 quantitative traits exhibited wide phenotypic variation across the panel (Supplementary Table S1; Supplementary Figure S1). The highest coefficients of variation were observed for useless seed mass (CV = 149.0%), stem diameter (CV = 110.0%), and plant canopy length (CV = 72.0%). The high variability in useless seed mass reflects strong differences among lines in the occurrence of poorly developed or non-usable seeds. Flowering and maturity traits showed moderate variation (CV = 14.0–18.0%), while pod and seed traits displayed intermediate to high variability, particularly pod width (CV = 66.0%) and weight of ten dry pods per plot (CV = 34.0%). Trait distributions supported these patterns (Supplementary Figure S1). Frequency distributions indicated that most traits were concentrated in a limited number of classes, with clear modal values. Emergence traits showed narrow distributions. Flowering traits followed nearly symmetric distributions centered on typical developmental stages. In contrast, pod and seed traits were right-skewed, with most lines showing lower values and only a small proportion showing high values. Shannon diversity indices (H′ = 1.53–3.09) indicated the highest diversity for yield-related traits, including 1000-seed mass (H′ = 3.09), weight of ten dry pods per plot (H′ = 2.88), and total seed mass (H′ = 2.78). Early-stage traits such as emergence and canopy length showed lower diversity (H′ < 1.8), consistent with more uniform early growth.

### 2.3. Qualitative Trait Frequencies and Diversity

The 37 qualitative morphological and stress-related traits also showed substantial variation among the 507 evaluated lines (Supplementary Figure S2). Flower color was mainly white, pink, or purple, while other color states occurred at low frequency. Most lines had triangular leaflets, and the indeterminate climbing growth habit was the most common architecture. Pod color and pod curvature at maturity were among the most variable traits, with several pigmentation types present at low to intermediate frequencies. In contrast, biotic stress symptoms such as anthracnose, rust, and virus-like patterns were rarely observed. Shannon diversity indices varied widely across traits. Pod color and pod curvature showed relatively high diversity (H′ ≈ 1.41 and 1.19), whereas disease- and pest-related traits showed low diversity (H′ < 0.3) due to the dominance of one or two states. Overall, pigmentation traits and pod morphology contributed most to qualitative diversity, while structural traits were more uniform.

### 2.4. Correlation Structure Among Quantitative Traits

**Figure 2:**
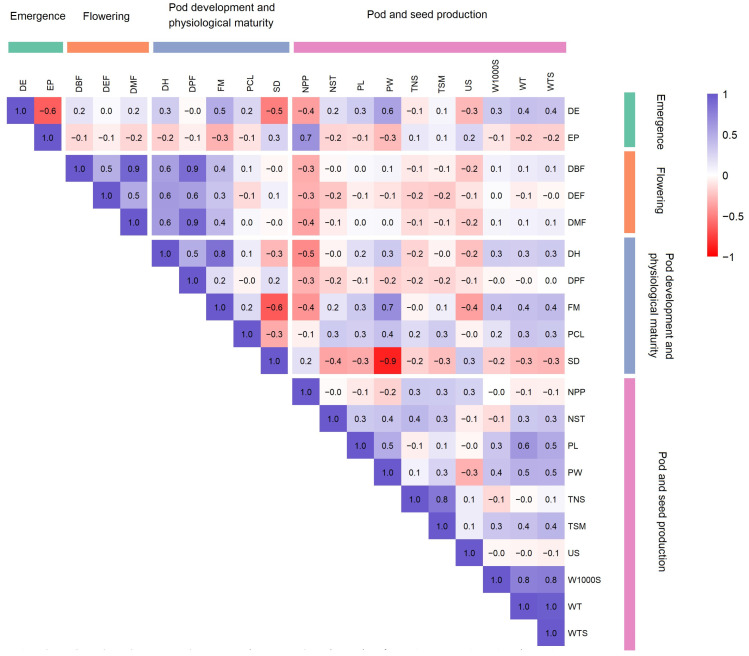
Correlation heatmap of 20 quantitative traits in T-core common-bean lines. DE: Days to emergence [days]; EP: Emerged plants [number]; DBF: Days to beginning of flowering [days]; DMF: Days to maximum flowering [days]; DEF: Days to end of flowering [days]; DPF: Days to pod formation [days]; FM: Full maturity [days]; DH: Days to harvest [days]; NPP: Number of plants with pods per plot [number]; PL: Pod length [cm]; PW: Pod width [cm]; WT: Weight of ten dry pods per plot [g]; NST: Number of seeds in ten dry pods per plot [number]; WTS: Weight of total seeds in ten dry pods per plots [g]; W1000S: 1000-seed mass [g]; TNS: Total number of seeds [number]; TSM: Total seed mass [g]; US: Useless seed mass [g]; PCL: Plant canopy length [cm]; SD: Stem diameter [mm]. Statistical significance of correlation coefficients was assessed, with |r| ≥ 0.3, 0.5, and 0.7 corresponding approximately to moderate, strong, and very strong associations, respectively.

The correlation matrix (Figure 2) revealed several clear relationships among traits. Flowering and maturity traits (DBF, DMF, DEF, DPF, FM, DH) were strongly and positively correlated (r = 0.30–0.90). Yield-related traits also showed coordinated behavior. Total seed mass (TSM) was strongly correlated with total seed number (TNS) (r = 0.80) and was positively associated with 1000-seed mass (W1000S) and weight of ten dry pods per plot (WT). Several negative correlations were observed. A negative correlation was found between stem diameter (SD) and pod width (PW) (r = −0.90). Stem diameter (SD) also showed a negative correlation with full maturity (FM) (r ≈ −0.10). Days to emergence showed negative correlations with emerged plants, stem diameter, pod-bearing plants per plot, and useless seed mass (r = −0.30 to −0.60).

### 2.5. Quantitative Trait Structure Revealed by PCA

**Figure 3:**
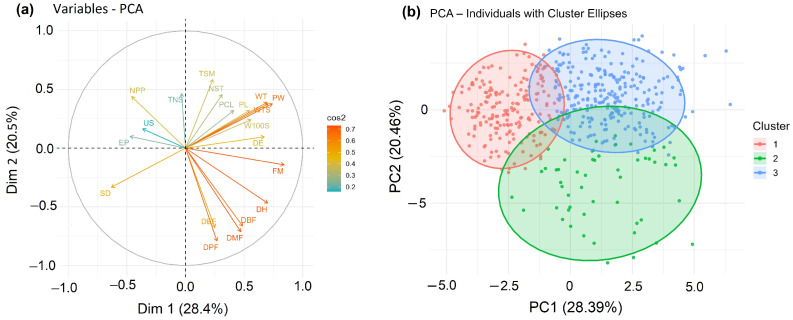
Principal Component Analysis (PCA) of 507 T-core common-bean lines based on 20 quantitative traits. (**a**) Variable loadings showing trait contributions of quantitative traits to the first two principal components. (**b**) Individual factor map showing the distribution of lines in the PC1–PC2 space, with ellipses indicating three broad quantitative phenotypic groups identified in the PCA space. The PCA highlights the main gradients of quantitative phenotypic variation within the panel.

Principal component analysis (PCA) showed that the first two components explained 48.9% of the total variation (PC1 = 28.4%; PC2 = 20.5%) (Figure 3). PC1 was mainly associated with pod and seed traits, including pod width, pod weight, total seed mass, and full maturity. PC2 captured variation in reproductive timing, driven by flowering and pod formation traits (DBF, DMF, DEF, and DPF). The PC1–PC2 scatterplot (Figure 3a) showed clear phenotypic gradients among lines. Lines ranged from early-flowering lines with small pods to intermediate types with moderate canopy growth and later-maturing lines with larger pods. These gradients formed the basis for subsequent mixed-data clustering.

### 2.6. Mixed-Data Ordination (FAMD) and HCPC Clustering

**Figure 4:**
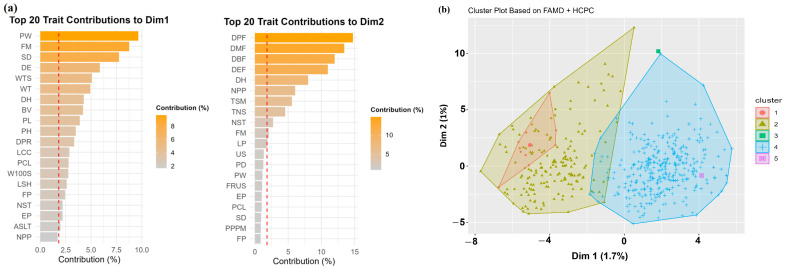
Factor Analysis of Mixed Data (FAMD) and HCPC clustering of 507 common-bean lines based on quantitative and qualitative traits. (**a**) Top 20 trait contributions to the first two FAMD dimensions. (**b**) Individual factor map showing five phenotypic clusters identified by Hierarchical Clustering on Principal Components (HCPC). The analysis summarizes the main mixed-trait structure of the panel.

Factor Analysis of Mixed Data (FAMD) was applied to the 507 lines with complete phenotypic data. The first two dimensions explained a small proportion of the total inertia (Dim1 = 1.7%; Dim2 = 1.0%). This indicates that mixed-trait variation was distributed across several dimensions rather than concentrated in a small number of dominant axes. The main gradients were consistent with those identified by PCA and MCA. Dim1 was mainly influenced by pod and seed morphology, with strong contributions from pod width, full maturity, stem diameter, days to emergence, total seed weight, and pod weight. Dim2 was driven by phenological traits related to flowering, pod formation, and harvest timing (Figure 4a).

#### 2.6.1. HCPC Clustering

Hierarchical clustering on the FAMD coordinates separated the lines into five phenotypic clusters (Figure 4b). The clusters differed in size, position, and dispersion. Cluster 4 was the largest and most compact group, occupying a central position. Clusters 1 and 2 were more dispersed and extended mainly along the negative side of Dim1. Clusters 3 and 5 each consisted of a single line and appeared as isolated points. These two clusters were retained in the classification because they represented distinct phenotypic profiles identified by the HCPC procedure.

#### 2.6.2. Statistical Validation of Cluster Differences

Analysis of variance showed that 19 of the 20 quantitative traits differed significantly among clusters (*p* < 0.001), except for days to end of flowering (DEF) (Supplementary Table S2). Tukey HSD comparisons supported these differences among cluster means (Supplementary Table S3). Cluster 4 generally showed the highest values for seed- and yield-related traits, including total seed mass, weight of ten dry pods per plot, number of seeds in ten dry pods per plot, weight of total seeds in ten dry pods per plot, 1000-seed mass, and total number of seeds. Clusters 1 and 2 were characterized by earlier flowering and lower seed mass. Clusters 3 and 5, each consisting of a single line, were interpreted as individual phenotypic extremes rather than as broader cluster types (Supplementary Table S3). These lines were retained in the classification because they represented distinct trait combinations identified by the HCPC procedure. Broadly similar patterns were also observed across biological status, geographic region, and growth habit. Landraces generally had larger canopy size and higher seed mass than cultivars, whereas hybrid and breeding or research materials tended to show lower values for most traits (Supplementary Table S3). Lines from Southern Europe and North Africa tended to have larger pod and seed traits, while Sub-Saharan African lines showed lower values for yield-related traits. Indeterminate climbing lines were associated with larger canopies and higher seed production, whereas determinate and indeterminate bush types generally had lower values. Full pairwise comparisons are provided in Supplementary Table S3.

### 2.7. Selection Index (SI)

**Figure 5:**
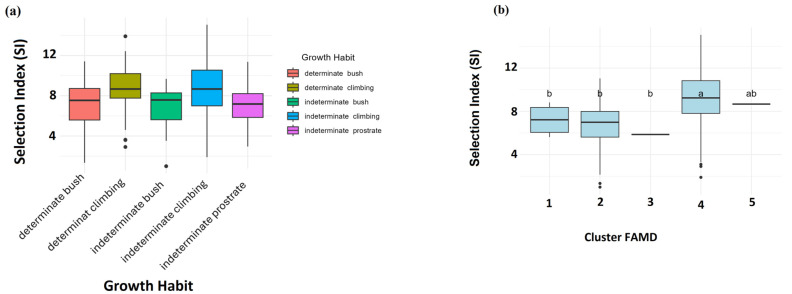
Selection Index (SI) variation across (**a**) HCPC clusters and (**b**) growth habits.

The multi-trait SI summarized agronomic performance across quantitative and qualitative traits. SI values ranged from 1.0 to 15.06 (mean 8.70 ± 2.90) and differed significantly among clusters (Figure 5a). The full SI dataset is provided in Supplementary Table S4, with the top 30 lines listed in Table 1. Cluster 4 had the highest SI values, while Clusters 1 and 2 had intermediate SI values (Figure 5a). The single-line clusters showed low SI in Cluster 3 and moderate SI in Cluster 5. All top-ranked lines, including INCBN_03268 (SI = 15.06), INCBN_02799 (14.84), and INCBN_01194 (14.51), belonged to Cluster 4. These were mainly indeterminate climbing landraces. SI values differed strongly among growth habits (Figure 5b), with climbing types ranking highest and bush or prostrate types showing lower values.

### 2.8. Qualitative Trait Structure Revealed by MCA

**Figure 6:**
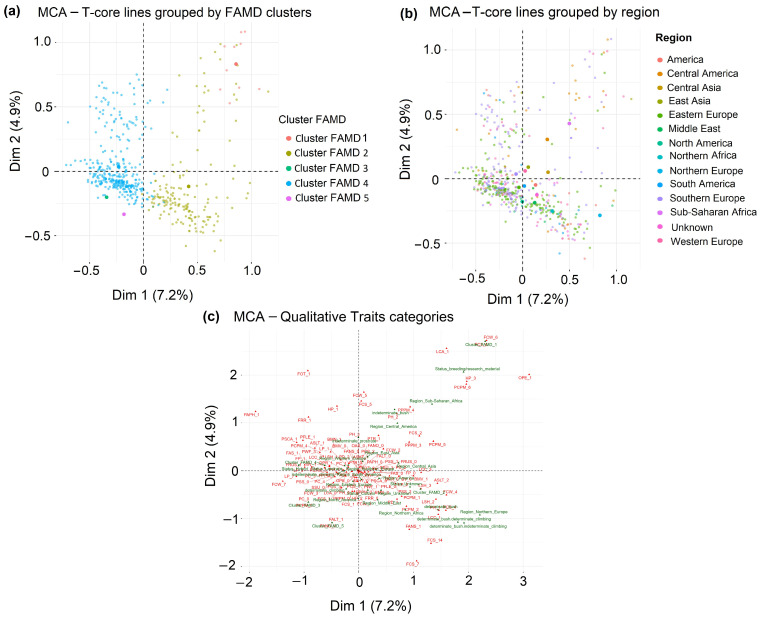
Multiple Correspondence Analysis (MCA) of qualitative traits in 507 common-bean T-core lines: (**a**) Individual factor map colored according to HCPC cluster membership, showing partial phenotypic separation among phenotypic groups. (**b**) Individual factor map colored by geographic region, indicating overlap among regions. (**c**) MCA variable map showing the qualitative traits categories contributing most strongly to the first two dimensions. Pigmentation-related traits contributed mainly to Dim1, whereas stress- and disease-related descriptors contributed more strongly to Dim2.

Multiple Correspondence Analysis (MCA) of qualitative traits explained 7.2% of the inertia on Dim1 and 4.9% on Dim2 (Figure 6a), indicating that the first two dimensions captured only a limited proportion of the total qualitative variation. When lines were colored by FAMD clusters, Cluster 4 appeared relatively compact near the origin, whereas Clusters 1 and 2 were more widely dispersed across the MCA space. Clusters 3 and 5, each represented by a single line, occupied peripheral positions. Coloring by geographic origin showed substantial overlap among regions, with no clear separation along the first two MCA dimensions (Figure 6b). Dim1 was mainly associated with pigmentation-related traits, whereas Dim2 was influenced more strongly by stress- and disease-related categories (Figure 6c). High-frequency states clustered near the origin, while rare morphological states contributed more strongly to dispersion. Overall, MCA provided a complementary view of qualitative variation, but with more limited resolution than the quantitative and mixed-data analyses.

### 2.9. Weather Conditions Across the Two Growing Seasons

The two growing seasons differed clearly in temperature and precipitation patterns (Supplementary Figure S3). Temperatures were similar between years during early growth, but 2022 remained slightly warmer in late summer and early autumn. Precipitation showed a stronger contrast. The 2021 season was relatively dry during the main reproductive period, whereas rainfall increased markedly in 2022, particularly in September. Relative humidity was also higher in 2022 during late-season stages. Together, these patterns indicate that the two growing seasons differed in their seasonal weather profiles.

### 2.10. Yield Performance and Year-to-Year Variation in a Subset of High-Performing Lines

The analyses above describe phenotypic diversity across 507 T-core lines evaluated in a single environment. To assess yield performance, stability, and year-dependent response, a subset evaluated in both years was required. Nineteen R-core lines met this criterion and were selected for year-to-year analysis. The following analyses are based on data collected at a single location over two growing seasons and therefore do not represent a formal multi-environment trial. The results reflect differences between years under the conditions of this site. All selected lines belonged to HCPC Cluster 4, which was generally characterized by indeterminate growth habit and large canopy size. The subset captured variation in growth habits (indeterminate climbing, indeterminate prostrate, and determinate climbing) and geographic origin (Southern, Western, and Eastern Europe), while biological status (landraces) remained uniform (Supplementary Table S5).

#### 2.10.1. AMMI Analysis of Yield Performance and Stability

**Figure 7:**
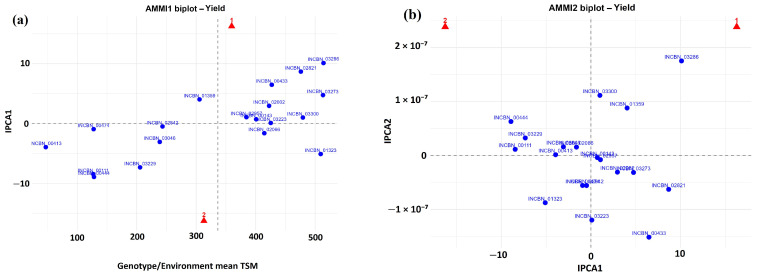
(**a**) AMMI1 and (**b**) AMMI2 biplots for yield (total seed mass, TSM) for 19 selected common-bean R-core lines across two years. AMMI1 shows mean yield (x-axis) and IPCA1 scores (y-axis), while AMMI2 displays interaction patterns based on IPCA1 and IPCA2. Genotypes closer to the origin indicate greater stability, whereas those farther from the origin show stronger interaction across years.

The AMMI1 biplot (Figure 7a) showed clear differences among lines for mean yield and IPCA1 scores. Lines located to the right of the grand mean, including INCBN_03286, INCBN_03273, INCBN_03300, INCBN_02821, and INCBN_02002, had above-average yield. Among these, INCBN_03273, INCBN_03300, and INCBN_02002 were positioned closer to the IPCA1 axis, indicating lower IPCA1 values. In contrast, lower-yielding lines such as INCBN_00413, INCBN_00111, and INCBN_00444 had larger IPCA1 scores. Determinate climbing lines, including INCBN_00111 and INCBN_00413, had both lower yield and higher interaction.

The AMMI2 biplot (Figure 7b) displayed the interaction structure based on IPCA1 and IPCA2. Lines located near the origin, including INCBN_00413, INCBN_00143, and INCBN_02957, had low interaction values. Several high-yielding lines, such as INCBN_03286, INCBN_03300, and INCBN_02821, were positioned farther from the origin. INCBN_01323 and INCBN_03223 occupied different quadrants of the biplot.

#### 2.10.2. Yield Performance, Stability and Yield-Stability Index (YSI)

Mean yield ranged from 46.99 g (INCBN_00413) to 514.05 g (INCBN_03286 and INCBN_03273) (Table 2). Stability metrics also varied greatly. Based on ASV/WAAS, the most stable lines were INCBN_03223 (2.25), INCBN_02842 (11.26), and INCBN_00143 (16.45). Highly stable lines such as INCBN_03223 and INCBN_00143 exhibited moderate yield levels. In contrast, several lines, including INCBN_03286, INCBN_01323, and INCBN_03273, had high yield but lower stability. When yield and stability were combined using the Yield–Stability Index (YSI), lower values indicated better performance. INCBN_03223 ranked highest (YSI = 8.00), followed by INCBN_03300 (YSI = 9.00) and INCBN_00143 (YSI = 13.00). All top-ranked YSI lines were indeterminate landraces.

### 2.11. GGE Biplot Analysis of Yield and Year Relationships

**Figure 8:**
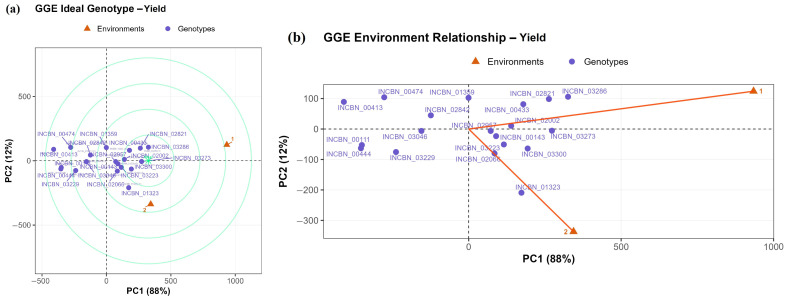
GGE biplot analysis of yield performance for 19 selected common-bean R-core lines evaluated across two years. Yield was measured as total seed mass (TSM). (**a**) Ideal-genotype view showing the relative performance and stability of genotypes based on the first two principal components. Genotypes positioned closer to the center of the concentric circles are considered more desirable in terms of combined yield performance and stability. (**b**) Year-relationship view showing the relative discriminating ability and contrast between the two growing seasons. Longer vectors indicate greater discriminating ability, whereas the angle between vectors reflects differences in genotype ranking between years.

Whereas AMMI was more informative for yield stability and interaction structure, the GGE biplot provided a clearer view of genotype ranking and the relative contribution of the two years. The GGE biplot for yield explained 88.0% of the variation in PC1 and 12.0% in PC2 (Figure 8a). PC1 primarily reflected differences in mean yield, while PC2 captured year-dependent interaction effects. Lines INCBN_03273, INCBN_02821, INCBN_03286, and INCBN_02002 were positioned closest to the ideal point. The year-relationship biplot (Figure 8b) showed a longer vector for 2021, indicating higher discriminating ability. The 2022 vector was shorter and displayed a different interaction pattern.

### 2.12. MGIDI Index and Multi-Trait Performance Across Years

**Figure 9:**
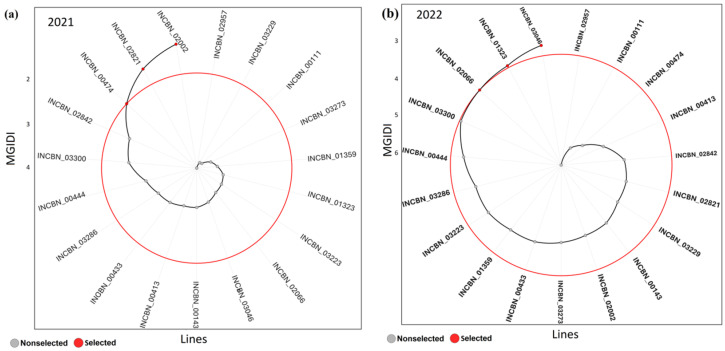

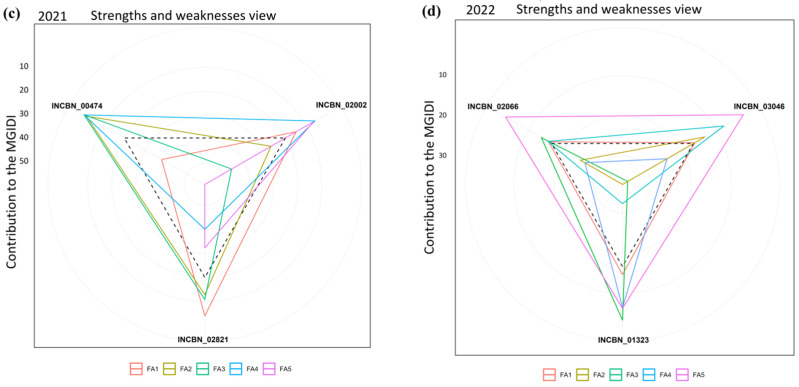
Multi-trait Genotype–Ideotype Distance Index (MGIDI) and factor-based strengths-weaknesses analysis of 19 selected common-bean R-core lines evaluated in 2021 and 2022. (**a**) MGIDI ranking in 2021 and (**b**) MGIDI ranking in 2022, based on 20 quantitative traits. Lower MGIDI values indicate genotypes closer to the ideotype and therefore more desirable overall performance. (**c**) Factor contribution plot for 2021 and (**d**) factor contribution plot for 2022, showing the relative strengths and weaknesses of individual genotypes across the retained factor structure. These panels illustrate how different trait groups contributed to genotype performance in each year.

The MGIDI index revealed clear differences in multi-trait performance between years (Figure 9). In 2021, only a limited number of lines fell within the selection boundary, with INCBN_02002, INCBN_02821, and INCBN_00474 showing the lowest MGIDI values (Figure 9a). In contrast, several lines, including INCBN_02842 and INCBN_03300, were positioned farther from the plot center. In 2022, a similar overall pattern was observed, although the ranking of some lines changed (Figure 9b). INCBN_03046, INCBN_01323, and INCBN_02066 ranked among the lines with the lowest MGIDI values, whereas INCBN_03300 remained distant from the center. Lines such as INCBN_02957 and INCBN_00111 consistently showed higher MGIDI values. Across both years, only a small subset of lines repeatedly exhibited low MGIDI values. Together, these analyses identified a subset of promising lines with favorable yield and multi-trait performance across the two evaluated seasons, supporting their prioritization for further validation.

### 2.13. Multi-Trait Structure and MGIDI Strengths-Weaknesses

Factor analysis grouped the quantitative traits into five factors in 2021, explaining 82.7% of the total variance (Table 3; Figure 9c). FA1 was dominated by phenology traits, with high loadings for days to flowering and pod formation. FA2 captured yield and canopy-related variation, with strong contributions from total seed mass, total seed number, pod and seed weights, and canopy length. FA3 mainly reflected emergence and stand traits, including days to emergence, emerged plants, and the number of pod-bearing plants. FA4 was associated with seed and pod size together with maturity timing, while FA5 was mainly driven by pod length and useless seed mass. These factor groupings underpinned the MGIDI strengths–weaknesses profiles in 2021. In 2022, six factors explained 87.5% of the total variance (Table 3; Figure 9d). FA1 again represented phenology, with high loadings for flowering, pod formation, maturity, and harvest timing. FA2 was dominated by emergence and plant size components, including days to emergence, emerged plants, canopy length, and stem diameter. FA3 captured overall yield outcome through total seed number and total seed mass. FA4 was driven by seed size and useless seed mass, whereas FA5 was mainly associated with pod and seed weight traits. Pod morphology was separated into FA6, with strong loadings for pod length and pod width. Compared with 2021 (Figure 9c), yield-related traits were partitioned into more distinct factors in 2022. The shift in factor structure between years was also reflected in MGIDI ranking. This suggests that the relative importance of trait groups differed between 2021 and 2022. Consequently, some lines ranked more favorably in one year than in the other, indicating that multi-trait performance depended partly on year-specific patterns of trait association.

## 3. Discussion

### 3.1. Phenotypic Diversity and Gene-Pool Structure in the INCREASE R-Core

This study characterizes the phenotypic diversity and structure of a wide subsample of the INCREASE common-bean R-core and evaluates its value as a reference panel for breeding and year-to-year analyses. The set analysed here consisted mostly of lines from INCREASE T-core, which was developed as a reduced subset of the full INCREASE collection. Despite its smaller size, it remains representative of the broader diversity and allows detailed phenotypic evaluation under field conditions. The analysis focuses on phenotypic expression observed in open-field trials, providing a practical perspective on diversity that is directly relevant for selection. Because the excluded lines did not reach flowering, they could not be evaluated for most of the phenotypic traits analyzed here. The diversity patterns described in this study therefore refer to the subset of lines that completed reproductive development under the field conditions of the trial. Wide variation was observed in phenology, plant architecture, and yield components. This supports the usefulness of field-based phenotyping for capturing complex trait expression and trade-offs under realistic agronomic conditions, as reported in previous studies on the common bean and other legumes [29,30].

The panel included lines ranging from small-seeded, early-maturing types to late, large-seeded ones, reflecting a wide spectrum of phenotypic diversity. The magnitude of variation observed is comparable to that reported in other diverse common-bean panels and landrace collections [2,31]. Seed size emerged as a major source of quantitative variation within the R-core and contributed strongly to the phenotypic structure revealed by multivariate analyses (Figure 4 and Figure 5). Traits related to seed size and associated reproductive characteristics aligned with axes of variation commonly linked to the Mesoamerican and Andean gene pools, indicating that signatures of these domestication backgrounds remain detectable at the phenotypic level. Rather than separating lines into sharply defined groups, these traits structured the collection along continuous multivariate gradients, consistent with historical introgression and recombination, particularly within European germplasm. Similar patterns of gradual differentiation have been reported in previous studies of common-bean diversity [6,32,33]. Overall, the diversity captured in the INCREASE collection likely reflects the combined influence of the two major gene pools, local farmer selection in landraces, and modern breeding activities, providing a useful phenotypic framework for identifying promising trait combinations for further evaluation.

Correlation analysis showed clear relationships between growth, yield, and yield-component traits (Figure 2). Total seed mass (TSM) showed a strong positive correlation with total seed number (TNS) and seed weight in ten pods (WTS). This suggests that variation in yield mainly reflected coordinated changes in its main components, as previously reported in the common bean [34]. In contrast, 1000-seed mass (W1000S) was negatively associated with traits related to seed number (NST), indicating that R-core lines with larger seeds tended to produce fewer seeds [35]. Pod and seed size traits (PL, PW, WTS, W1000S) showed positive correlations (Figure 2). Pod morphology and seed dimensions therefore increased together, as observed in other common-bean diversity studies [9,31]. A strong negative correlation was observed between stem diameter (SD) and pod width (PW) (r ≈ −0.9). However, this pattern appeared to reflect the separation of contrasting phenotypic groups within the panel rather than a general physiological trade-off across all lines. In diverse germplasm collections, such correlations may arise from structured phenotypic differentiation rather than direct trait-to-trait biological dependence. PCA summarized the relationships among quantitative traits into two main axes of variation (Figure 3). The first principal component was mainly associated with reproductive organ size and maturity-related traits, separating earlier and smaller genotypes from later-developing lines with larger pods and seeds. The second component was driven primarily by yield components and canopy-related traits, including total seed mass, total seed number, and canopy length, reflecting differences in overall reproductive productivity. Together, these axes indicate that phenotypic variation within the INCREASE R-core is structured along gradients of developmental timing, reproductive size, and yield potential, rather than forming discrete phenotypic groups. Similar patterns of continuous variation have been reported in diverse common-bean germplasm collections [5,32,33].

### 3.2. Integrated Phenotypic Structure Revealed by FAMD-HCPC

To integrate quantitative and qualitative traits, FAMD was applied, followed by HCPC. As expected for large mixed datasets, the first FAMD dimensions explained only a limited proportion of the total inertia. This is common in mixed datasets with many descriptors, where variation is usually spread across several dimensions rather than concentrated in the first few axes. In the present panel, this pattern likely reflects both the complexity of the trait set and the partial overlap of phenotypic profiles among lines with different biological and geographic backgrounds. Despite the low inertia of the first dimensions, the main gradients were biologically interpretable and consistent with those identified by PCA and with patterns reported in previous common-bean diversity studies [9,31,36]. The first FAMD dimension primarily described variation in plant size and yield components, while the second dimension captured phenological differences from early to late flowering and maturity. HCPC was therefore interpreted in relation to cluster trait profiles, its agreement with the other multivariate analyses, and the observed differences among clusters for most quantitative traits. Taken together, these patterns suggest that the clustering captured biologically meaningful phenotypic structure.

HCPC separated the lines into five phenotypic clusters with distinct agronomic profiles. One large and clearly defined cluster (Cluster 4) included high-yielding, large-seeded, mostly climbing R-core lines (Figure 4). This profile resembles Andean-type ideotypes described in earlier studies [5,8,33,37]. Other clusters represented earlier and smaller-seeded types. Two clusters consisted of single lines with extreme trait combinations. Such single-line clusters are not unusual in multivariate analyses and likely reflect isolated phenotypic extremes rather than stable or broadly representative population groups [38,39]. Although they are not informative as cluster types, such lines may still be useful when their extreme trait combinations are of interest for specific breeding objectives. The differentiation among HCPC clusters was also supported by univariate comparisons of quantitative traits. Most traits differed significantly among clusters, whereas flowering duration showed much less separation. Similar validation approaches have been widely applied in recent common-bean diversity studies [10,31,36].

### 3.3. Biological Status and Regional Patterns

Grouping lines by biological status (landrace, cultivar, breeding or research line, hybrid, unknown) provided additional insight into the structure of phenotypic diversity, as most R-core lines belonged to the landrace category. Landraces were widely distributed across clusters, ranging from early, small-seeded types to large-seeded climbing forms. This broad distribution reflects their high internal diversity and the combined effects of local adaptation and farmer selection [5,40].

Cultivars formed more compact groups, with intermediate pod and seed size and relatively stable yield. This pattern is consistent with selection for uniformity and market requirements [6,35,41]. Breeding lines and hybrids were each represented by a single line and were therefore considered individual cases rather than indicators of broader patterns.

Regional differences broadly followed the main multivariate patterns. Lines associated with Southern Europe, North America, and North Africa generally had larger pods and higher seed mass. In contrast, lines from Sub-Saharan Africa tended to have smaller pods and seeds. This pattern agrees with earlier regional comparisons reported in international bean collections [36,37,42]. Growth habit again emerged as an important factor. Indeterminate climbing types had the largest canopies and the highest total seed number and seed mass. In comparison, determinate and indeterminate bush types consistently showed lower values [42,43,44].

Previous studies have shown that phenotypic clustering does not always correspond directly to molecular population structure [32,44]. Our results provide a complementary, phenotype-based view of diversity that is particularly relevant for agronomic evaluation. Similar phenotypes can arise through convergent selection rather than shared ancestry. For this reason, the clusters identified here should be interpreted mainly as functional agronomic groupings, not as direct representations of genetic population structure.

### 3.4. Multi-Trait Selection Indices as Tools to Identify Ideotypes

To summarize information across multiple traits, a Selection Index (SI), based on classical SI theory [20] and recent approaches such as MGIDI [22], was used. Such indices are increasingly applied in self-pollinated crops, including the common bean, to rank R-core lines while accounting for correlations and trade-offs among traits. The SI weights were assigned to reflect the agronomic importance of the measured traits and their expected contribution to overall performance. This introduced a degree of subjectivity, but it allowed the index to emphasize traits considered more relevant for selection in the present dataset. In contrast, MGIDI was calculated directly from the multivariate structure of the phenotypic data and did not require predefined trait weights. The broad agreement between the two approaches suggests that the main ranking patterns were reasonably stable. In our study, the SI clearly identified R-core lines that combined high yield with favorable pod and seed traits and suitable phenology. Cluster 4, previously identified as the high-yielding and large-seeded group in the HCPC analysis, also showed the highest mean SI values (Figure 5a). This further supports its relevance as a source of promising phenotypic profiles [22,29]. Early, small-seeded clusters showed much lower SI values, consistent with the known relationship between plant size and yield potential in the common bean [28]. Single-line clusters showed extreme SI values but should be treated as outliers rather than broadly useful material [38,39]. The SI values differed strongly among growth habits, with the highest values in indeterminate climbing types and the lowest in determinate bush types. These results reinforce the importance of plant architecture for yield potential. Previous studies have shown that climbing ideotypes often use resources more efficiently and achieve higher yields under favorable conditions [33,43]. The overall ranking pattern was broadly consistent with the multi-trait trends identified by MGIDI in the two-year subset analysis.

### 3.5. Qualitative Trait Diversity and Role of MCA

Multiple Correspondence Analysis (MCA) of 37 qualitative descriptors showed that only a limited number of trait states contributed strongly to qualitative differentiation. The first MCA dimensions explained only a small proportion of the total inertia, which is typical for MCA applied to morphological datasets [45,46]. This indicates that qualitative variation in the panel was distributed across many descriptors, with no small set of dimensions capturing a large share of the overall structure. Most common states clustered near the origin, indicating that many standard descriptors had limited discriminatory power for separating lines, as often observed when frequent categories discriminate the dataset [47]. In contrast, rarer or more extreme states contributed more strongly to the observed dispersion. This pattern was especially evident for pigmentation traits and for stress- or disease-related symptoms. Similar findings have been reported in other bean diversity studies using MCA [9,31]. Common classes for flower traits, pod traits, and growth habit largely overlapped in the center of the MCA space, suggesting that these traits are widely shared across the collection and therefore contributed limited resolution for line discrimination. Region and biological status also showed weak structure in the MCA, indicating that qualitative diversity was not strongly partitioned by these external factors. MCA therefore served mainly as a complement to PCA and FAMD–HCPC, helping to identify a smaller set of uncommon morphological and stress-related descriptors rather than defining the main structure of phenotypic diversity.

The analysis of the 507 R-core lines provided a broad view of the phenotypic diversity in the collection. It also clarified how major traits such as seed size, growth habit, and yield components are structured across the panel. These results help describe the general variation in the R-core, but they do not show how lines respond across years under differing seasonal conditions or how stable their performance is across seasons. To explore these questions, we used a smaller set of 19 lines that were evaluated in both years. This subset represents only part of the full diversity, but it includes clear contrasts in growth habit and seed size. These features make this subset suitable for linking overall phenotypic structure with the environmental responses of selected lines. The following section examines the performance of these 19 lines across two years and describes how their multi-trait profiles changed under different field conditions.

### 3.6. Yield Stability and Year-to-Year Variation Across Two Growing Seasons

All 19 selected lines belonged to the dominant phenotypic group identified in the first part of the study (HCPC Cluster 4). They shared key structural features, including landrace biological status, predominantly indeterminate climbing or prostrate growth habits, and origins mainly from Southern and Eastern Europe. This subset represents the high-performing ideotypic space within the R-core rather than the full phenotypic spectrum.

Despite their shared cluster membership and similar phenotypic profiles under single-environment evaluation, clear year-dependent interaction in yield was detected using both AMMI and GGE analyses (Figure 7 and Figure 8). Such interaction effects are well documented in the common bean and other self-pollinated legumes and reflect differential sensitivity of genotypes to seasonal variation [14,48,49]. These results indicate that substantial differences in yield stability and year-dependent response can arise even within a phenotypically coherent and high-performing group. The two approaches were informative in slightly different ways. AMMI was more useful for identifying differences in yield stability among lines, whereas GGE provided a more direct visual summary of genotype ranking and year-specific discrimination.

In the AMMI1 biplot, several lines combined above-average yield with low IPCA1 scores, indicating a balance between productivity and stability across years. INCBN_03273, INCBN_03300, and INCBN_02002 were positioned close to the IPCA1 axis while exceeding the grand mean yield (Figure 7a), suggesting more consistent performance across the two growing seasons. Similar patterns have been reported in multi-location and multi-year studies of the common bean [29,50]. In contrast, lower-yielding lines such as INCBN_00413 and INCBN_00111 showed larger IPCA1 values, indicating greater sensitivity to environmental variation, a pattern frequently observed in legume multi-environment trials [14,51]. The AMMI2 biplot further resolved the interaction pattern by jointly considering IPCA1 and IPCA2 (Figure 7b). Lines located near the origin, including INCBN_00413, INCBN_00143, and INCBN_02957, showed low interaction effects but also relatively low mean yield. In contrast, high-yielding lines such as INCBN_03286, INCBN_03300, and INCBN_02821 were positioned farther from the origin, indicating stronger interaction effects and possible specific adaptation. The contrasting positions of INCBN_01323 and INCBN_03223 suggest crossover interaction between years. Such patterns are common in the common bean and are often associated with differences in seasonal temperature, and water availability [14,16,48]. In the present study, the two growing seasons differed mainly in precipitation pattern and late-season temperature, with 2022 showing higher rainfall during the reproductive phase. These seasonal differences likely contributed to the crossover responses and shifts in genotype ranking observed between years.

These trends were consistent with the GGE biplot analysis. In the GGE biplot, PC1 explained most of the variation (88.0%) and mainly reflected differences in mean yield, while PC2 (12.0%) captured year-dependent interaction effects (Figure 8a). INCBN_03273, INCBN_02821, INCBN_03286, INCBN_03300, and INCBN_02002 were positioned closest to the ideal genotype, indicating high yield combined with relatively low interaction across years [49,52]. The year-relationship biplot (Figure 8b) showed that the 2021 season had stronger discriminating ability, whereas 2022 contributed less to genotype separation and exhibited a different interaction pattern. The wide angle between the year vectors supports the presence of crossover interaction and indicates that the two seasons ranked lines differently. Similar seasonal contrasts have been widely reported in the common-bean field evaluations and highlight the importance of multi-year testing for a more reliable assessment of yield stability [29,48,50].

Beyond mean yield patterns, stability metrics provided additional resolution among elite materials. Although Cluster 4 was identified as a reservoir of favorable trait combinations based on single-environment phenotyping, year-to-year analyses revealed further differentiation among these lines for yield stability and adaptation. This highlights the value of incorporating year-to-year response analyses into phenotypic characterization when follow-up evaluation is possible and indicates that ideotype identification based on trait structure benefits from complementary stability assessment before breeding or recommendation decisions are made [29].

While AMMI and GGE analyses evaluated yield stability using total seed mass, the MGIDI index provided a complementary multi-trait assessment based on all quantitative traits. Only a small subset of lines consistently showed low MGIDI values across both seasons, with INCBN_03300, INCBN_00444, and INCBN_03223 ranking closest to the ideotype. Several high-yielding lines had higher MGIDI values, indicating that strong yield performance alone did not guarantee balanced multi-trait profiles. This divergence highlights the importance of accounting for trait correlations and trade-offs when defining elite materials [22]. Analysis of MGIDI strengths and weaknesses further showed that phenology-related traits contributed consistently to the factor structure across years, whereas pod, seed, and yield components were more variable and environment-dependent. This pattern suggests that multi-trait performance was not governed by exactly the same trait combinations in both years. In other words, changes in trait relationships across seasons influenced how closely each line approached the ideotype. Together, these results show that MGIDI complements year-based yield analyses by revealing trait-level limitations underlying yield stability and helps prioritize lines on the basis of multi-trait balance rather than yield alone.

Overall, the combined analysis of phenotypic diversity, mixed-trait structure, and year-to-year performance indicates that the INCREASE R-core sample captures both broad diversity and agronomically relevant differentiation among lines. The convergence of results from PCA, FAMD–HCPC, MCA, AMMI, GGE, and multi-trait selection indices links trait architecture with yield formation and environmental response in a coherent framework. By integrating large-scale single-environment phenotyping with focused year-to-year evaluation, this study illustrates how representative subsets can help descriptive diversity analyses and breeding-oriented interpretation. In this context, the R-core subsample provides a useful reference population for ideotype-oriented breeding, and the identification of promising lines for future replicated validation within the INCREASE initiative. From a breeding perspective, the results help identify a smaller set of priority lines within the broader panel. In the single-environment screening, several high-ranking SI lines from Cluster 4, including INCBN_03268, INCBN_02799, INCBN_01194, INCBN_03277, and INCBN_00496, emerged as promising material for further evaluation. Within the two-year subset, INCBN_03286, INCBN_03273, INCBN_03300, INCBN_02002, INCBN_03223, and INCBN_00143 appeared particularly relevant, depending on whether the main breeding objective is high yield, yield stability, or more balanced multi-trait performance. Across both levels of analysis, total seed mass, total seed number, pod and seed weight, and stable reproductive performance emerged as the most relevant target traits.

### 3.7. Study Limitations and Future Directions

The present study provides a broad phenotypic characterization of the INCREASE common-bean panel. It identifies major patterns of variation, trait associations, and promising lines with favorable multi-trait performance. At the same time, some aspects of the observed variation could not be fully resolved within the scope of the current design and should be addressed in future work. The main phenotypic evaluation was conducted at a single location and without replicated plots for each line. This design was suitable for large-scale comparative screening. However, it limits the extent to which genotypic differences can be separated from environmental and micro-environmental effects. The results also point to a broader biological question. Clear phenotypic structure was detected, but its relationship with stability and multi-trait performance was not always consistent across analyses and years. For example, differences in MGIDI ranking and factor structure between seasons suggest that the contribution of individual traits to overall performance can shift under different environmental conditions. This indicates that ideotype identification based on single-environment phenotypic structure captures only part of the variation relevant for adaptation. The subset of lines evaluated across two years provided useful insight into year-to-year variation within the phenotypically superior group. However, it also showed that performance differences can remain substantial even among lines with similar overall phenotypic profiles. This highlights the importance of further validation beyond single-location screening. Future work should therefore focus on replicated multi-location and multi-year evaluation of the most promising lines identified here. Such experiments would allow a more robust assessment of stability, adaptation, and trait consistency across years. Integration of phenotypic data with genomic and environmental information would further strengthen the value of the INCREASE panel for breeding and ideotype development.

## 4. Materials and Methods

### 4.1. Plant Material and Experimental Design

This study was conducted within the framework of the INCREASE project and focused on a subset of Single Seed Descent (SSD) lines of the Reference Core (R-core) of common bean (*Phaseolus vulgaris* L.) [24]. This subset comprised 641 lines, each assigned a unique Digital Object Identifier (DOI) to ensure full traceability (Supplementary Table S6). Plant materials were developed starting from heterogeneous lines provided by several international partner institutions, including the Centre national de la recherche scientifique (CNRS; Paris and South France), the Leibniz Institute of Plant Genetics and Crop Plant Research (IPK, Germany), the United States Department of Agriculture Western Regional Plant Introduction Station (Pullman, WA, USA) and the International Centre of Tropical Agriculture (Palmira, Colombia). The experiment was conducted over two growing seasons (2021 and 2022). In total, 447 lines were evaluated in 2021 and 194 in 2022 (Supplementary Table S6), with a subset of lines grown in both years. Of the 641 lines initially planted, 507 reached flowering and produced complete phenotypic data and were therefore included in diversity and multivariate analyses (Supplementary Table S6A). Lines that failed to reach reproductive development were excluded; a complete record of excluded material and data-handling procedures is provided in the Supplementary Information (Supplementary Table S6B).

Field experiments were conducted under open-field conditions at the experimental station of the Agricultural Institute of Slovenia in Jablje, Slovenia (46.1453° N, 14.5579° E; 302 m a.s.l.). The experimental site is characterized by eutric brown soil formed on older clay alluvium with gleyic features. Sowing was performed on 27 May 2021 and 20 May 2022. Each line was grown on two wooden poles placed 1 m apart. Sowing was carried out at three sowing points per pole, with seeds arranged in a circular pattern approximately 20–30 cm from the pole, at a depth of 2–4 cm using a hand-operated seed transplanter (BAZUKKA 2021). Ten seeds were sown per line (five per pole). Poles were positioned within black polyethylene-mulched rows (~1 m wide), with 2 m spacing between rows. The trials spanned two consecutive growing seasons (2021–2022), with each season considered a separate analytical unit representing contrasting seasonal conditions at the same location. The experiment followed a randomized single-row design. Each experimental unit consisted of one line sown around two support poles (five seeds per pole; ten seeds per line in total). Lines were randomized within the field in a single-plot layout and grown under the same agronomic conditions. Because the study was intended as a large-scale exploratory phenotyping trial, it was conducted without replication or formal blocking. Germination rates reached 93.0% in 2021 and 99.0% in 2022. Observations were conducted three times per week from emergence to maturity. Approximately 77.0% of the lines were successfully harvested, dried, and stored at −20 °C for post-harvest analyses. Phenotyping was performed according to standardized INCREASE descriptors.

Across both growing seasons, 19 lines produced complete yield data in both years and were included in year-to-year analyses of yield performance, yield stability, and year-dependent performance patterns. All diversity and multivariate analyses were performed on the full set of lines with complete phenotypic data for each year, whereas yield stability and year-to-year comparative analyses were restricted to lines evaluated in both seasons. Lines were grown in a single-replicate layout, in line with the exploratory objectives of INCREASE core collection phenotyping. Accordingly, analyses focused on phenotypic patterns, multivariate structure, and relative performance patterns among lines. Estimation of genetic variance components or heritability was not considered. Each plot corresponded to a single line and constituted the experimental unit. Standard agronomic practices for common-bean production in the region were applied consistently in both years. Weather data (monthly mean temperature, relative humidity, precipitation, and solar radiation) were obtained from the on-site meteorological station at the Jablje experimental field for the 2021 and 2022 growing seasons (Supplementary Figure S3).

### 4.2. Phenotyping of Agro-Morphological Traits

Phenotypic characterization was conducted using 57 agro-morphological traits, including 20 quantitative and 37 qualitative traits. Quantitative traits included emergence and flowering traits (days to emergence [DE], emerged plants [EP], days to beginning of flowering [DBF], days to maximum flowering [DMF], days to end of flowering [DEF]); pod development and physiological maturity traits (days to pod formation [DPF], full maturity [FM], days to harvest [DH]); plant architecture traits (plant canopy length [PCL], stem diameter [SD]); and pod and seed production traits (number of plants with pods [NPP], pod length [PL], pod width [PW], weight of ten dry pods per plot [WT], number of seeds in ten dry pods per plot [NST], weight of total seeds in ten dry pods per plot [WTS], 1000-seed mass [W1000S], total number of seeds [TNS], total seed mass [TSM], and useless seed mass [US], defined as the mass of unfilled, malformed, or otherwise non-usable seeds).

Qualitative traits described pigmentation and morphology (hypocotyl pigmentation [HP], leaf color based on chlorophyll [LCC] and anthocyanin [LCA] content, flower color of the standard [FCS] and wings [FCW], pod cross-section [PCS], pod curvature [PC], pod color at physiological maturity [PCPM], pattern of pod pigmentation at physiological maturity [PPPM], pod wall fiber [PWF], leaf persistence [LP], leaf shape [LSH], pod color on fully expanded immature pods [PCFE], and pod suture string at technological maturity [PSS]). Additional qualitative descriptors included plant determinacy [PD], plant growth habit [PH], and biotic and abiotic response traits covering disease presence [DPR], stress susceptibility [SSU], abiotic stress responses to low [ASLT] and high temperature [ASHT], other abiotic stress [OAS], pest incidence (thrips [PTR], aphid [PAPH], flea beetle [PFLE], *Scaphoideus titanus* [PSCA], and other pests [OPE]), fungal diseases (fungi present [FP], anthracnose [FANS], root rot [FRR], ascochyta [FAS], rust [FRUS], angular leaf spot [FANO], alternaria [FALT], and other fungi [FOT]), and bacterial and viral symptoms [BV], including bean common mosaic necrosis virus [BMN] and bean common mosaic virus [BMV]. All traits were recorded at the appropriate phenophase following standardized descriptor definitions for the common bean [24]. Full descriptor descriptions and scoring scales are provided in Supplementary Tables S7A,B.

### 4.3. Data Analysis

All analyses were conducted in R statistical software (version 4.3.1). Data visualization for weather was performed in R using *ggplot2* and patchwork. Qualitative traits were summarized using frequency distributions, and phenotypic diversity was quantified using the Shannon-Weaver diversity index (H′) [53]. Shannon indices were calculated with the *vegan* package. Variability of quantitative traits was summarized using the coefficient of variation (CV), calculated as the ratio of the standard deviation to the mean [54], using base R functions. Frequency distributions of quantitative traits were generated with *ggplot2*, following data manipulation with *dplyr* and *tidyr*. Associations among quantitative traits were evaluated using Pearson’s correlation coefficient after inspection of trait distributions [55], and correlation matrices were visualized as heatmaps using the *corrplot* and *pheatmap* packages. To summarize major axes of variation among quantitative traits, Principal Component Analysis (PCA) was applied to standardized data. Trait loadings and individual scores were used to interpret component structure and line distribution. PCA was implemented using *FactoMineR*, with graphical outputs generated using *factoextra* [46]. Quantitative and qualitative traits were jointly analyzed using Factor Analysis of Mixed Data (FAMD) implemented in the *FactoMineR* package, which allows integration of continuous and categorical variables within a single multivariate framework. The resulting phenotypic structure was further investigated by applying Hierarchical Clustering on Principal Components (HCPC) to the FAMD coordinates, using Ward’s minimum variance method [46]. Differences in quantitative traits were assessed across FAMD–HCPC clusters, geographic regions, biological status categories (landrace, cultivar, and breeding or research material), and growth habits using one-way analysis of variance (ANOVA). When significant effects were detected, means were separated using Tukey’s honestly significant difference (HSD) test. Residuals were checked for approximate normality and homogeneity of variance using Shapiro–Wilk and Levene’s tests, respectively. Because some traits showed deviations from these assumptions and some groupings were unbalanced, the Analysis of Variance (ANOVA) results were interpreted cautiously and used mainly to support broader phenotypic patterns. All analyses were conducted in R using base statistical functions and the *agricolae* package.

The selection index (SI) was based on classical selection index theory [18,20] and adapted to include penalties for stress and disease traits. The index was calculated as:

I*_i_* = b_1_x*_i_*_1_ + b_2_x*_i_*_2_ + … + b_p_x*_i_*_p_

where x_ij_ represents the phenotypic value of the *j*-th trait for line *i*, and b_j_ is the corresponding weight reflecting the relative importance of the trait. Trait weights were assigned based on breeding relevance, with higher positive weights given to yield and yield-component traits, moderate weights to plant vigor traits, and negative weights applied to late phenology, unfilled seeds, and stress- or damage-related traits. The full list of traits included in the SI, together with their assigned weights and brief breeding-based justification, is provided in Supplementary Tables S8A,B. Quantitative traits were standardized and combined using positive or negative weights according to breeding relevance. Binary qualitative traits were used as penalty terms, while ordinal traits were standardized and included with lower relative weights. Calculations and visualizations were conducted in R using *dplyr*, *FactoMineR*, and *factoextra*. Multiple Correspondence Analysis (MCA) was used to explore relationships among qualitative traits using *FactoMineR*, with visualization in *factoextra*. Qualitative traits were included as active variables, while Region, Biological Status, Growth Habit, and Cluster FAMD were treated as supplementary variables. The analysis was conducted on lines with complete qualitative data, and interpretation focused on individual distributions and category contributions to the first MCA dimensions [46]. MCA was used to explore associations among qualitative descriptors, complementing the mixed-data structure captured by FAMD.

### 4.4. Yield Performance and Stability Across Two Years

Yield performance and stability were evaluated using a subset of 19 common-bean lines assessed in both growing seasons. These lines were selected because they had complete and comparable phenotypic data in both years. The subset was therefore defined by data availability across years and was used to examine yield stability and multi-trait consistency within this narrower group of lines. Year-dependent response was evaluated using total seed mass (TSM), which was treated as the yield variable, with each year treated as a separate analytical unit representing contrasting seasonal conditions at the same location. Interaction patterns across years were explored using the Additive Main Effects and Multiplicative Interaction (AMMI) model [56], implemented in the *metan* R package [56].

AMMI1 and AMMI2 biplots were generated in R using the *metan* package, with graphical outputs prepared in *ggplot2*. Yield stability was assessed using the AMMI Stability Value (ASV) proposed by Purchase et al. [57]. ASV was used because it provides a simple numerical estimate of stability based on the first two interaction principal component axes from the AMMI model. In addition, the Weighted Average of Absolute Scores (WAAS) was calculated, because it incorporates all retained interaction axes. WAAS was further combined with mean yield to calculate the Yield–Stability Index (YSI) [22,58]. Lower ASV, WAAS, and YSI values indicate stable or more desirable genotypes, depending on the metric. Genotype plus Genotype × Environment (GGE) biplot analysis was also performed [16,52]. AMMI was used mainly to describe year-dependent interaction patterns and to identify relatively stable or unstable genotypes using AMMI-based stability statistics. In contrast, GGE was used to visualize genotype ranking across years and to examine how the two test years differed in their ability to discriminate among genotypes. Ideal-genotype and year-relationship views were used for interpretation. In addition to yield-based analyses, multi-trait performance was evaluated using the Multi-trait Genotype–Ideotype Distance Index (MGIDI) based on 20 quantitative traits following factor analysis, as implemented in the *metan* package [22]. The number of factors retained for MGIDI analysis was determined separately for each year using the eigenvalue > 1 criterion from factor analysis of standardized quantitative traits. This resulted in five factors in 2021 and six in 2022. Because the relationships among traits differed slightly between years, the factor structure was allowed to vary between datasets rather than being forced to remain identical. Comparisons between years were therefore based on overall genotype ranking, trait grouping, and relative distance to the ideotype, rather than on direct equivalence of individual factors. The MGIDI for the *i*-th genotype was calculated as:

$${\mathrm{MGIDI}}_{i}\;=\;\sqrt{\sum_{j=1}^{f}{\left(F_{ij}-F_{j}^{*}\right)}^{2}}$$

where *f* is the number of retained factors, F_ij_ is the score of genotype *i* for the *j*-th factor, and F_j_* is the ideotype score. Lower MGIDI values indicate genotypes closer to the ideotype. Factor contributions were used to identify strengths and weaknesses of individual genotypes [11,58].

## 5. Conclusions

The INCREASE common-bean R-core subsample evaluated in the present study captures a broad range of phenotypic diversity while retaining a clear and agronomically interpretable structure. Analysis of quantitative traits, qualitative descriptors, and mixed-trait data revealed the main sources of variation related to plant architecture, phenology, and yield formation, allowing the identification of distinct and functionally meaningful phenotypic groups. A dominant phenotypic cluster, largely composed of indeterminate climbing landraces, emerged as a useful source of favorable trait combinations. When a subset of these lines was evaluated across two growing seasons, clear year-to-year differences in performance were observed, even within this elite group. While some lines consistently combined high yield with stability, others showed pronounced sensitivity to seasonal conditions, highlighting the need for multi-year evaluation in addition to single-environment phenotyping. The partial agreement among AMMI, GGE, and multi-trait selection indices suggests that yield stability and ideotype-related performance are shaped by multiple, partly independent trait dimensions. Overall, the analytical framework applied here links phenotypic diversity with trait integration and year-dependent response. The INCREASE R-core therefore provides a useful reference population for ideotype-oriented breeding, the identification of lines with more consistent or more year-specific performance, and future integration of phenotypic, genomic, and environmental data aimed at improving common-bean adaptation and productivity.

## Figures and Tables

**Table 1:** Top 30 lines ranked by the Selection Index (SI).

Line	Growth Habit	Geographic Region	Country	Biological Status	Selection Index	SI Rank	HCPC Cluster
INCBN_03268	Indeterminate climbing	Eastern Europe	Slovakia	Landrace	15.06	1	4
INCBN_02799	Indeterminate climbing	Southern Europe	Italy	Landrace	14.84	2	4
INCBN_01194	Indeterminate climbing	Eastern Europe	Albania	Landrace	14.51	3	4
INCBN_03277	Indeterminate climbing	Eastern Europe	Slovakia	Landrace	14.35	4	4
INCBN_00496	Indeterminate climbing	Southern Europe	Spain	Landrace	14.30	5	4
INCBN_01417	Indeterminate climbing	Southern Europe	Croatia	Landrace	14.17	6	4
INCBN_02052	Indeterminate climbing	Eastern Europe	Georgia	Landrace	14.12	7	4
INCBN_00514	Indeterminate climbing	Middle East	Turkey	Cultivar	14.04	8	4
INCBN_02759	Indeterminate climbing	Southern Europe	Greece	Landrace	14.03	9	4
INCBN_01337	Indeterminate climbing	Western Europe	Austria	Landrace	13.94	10	4
INCBN_01203	Determinate climbing	Eastern Europe	Albania	Landrace	13.91	11	4
INCBN_01808	Indeterminate climbing	Eastern Europe	Georgia	Landrace	13.80	12	4
INCBN_01423	Indeterminate climbing	Southern Europe	Croatia	Landrace	13.33	13	4
INCBN_02933	Indeterminate climbing	Southern Europe	Italy	Landrace	13.33	14	4
INCBN_03286	Indeterminate climbing	Eastern Europe	Slovakia	Landrace	13.31	15	4
INCBN_01313	Indeterminate climbing	Western Europe	Austria	Landrace	13.22	16	4
INCBN_01335	Indeterminate climbing	Western Europe	Austria	Landrace	13.18	17	4
INCBN_01241	Indeterminate climbing	Eastern Europe	Albania	Landrace	13.09	18	4
INCBN_01199	Indeterminate climbing	Eastern Europe	Albania	Landrace	13.08	19	4
INCBN_00505	Indeterminate climbing	Southern Europe	Spain	Landrace	12.91	20	4
INCBN_00489	Indeterminate climbing	Eastern Europe	Bulgaria	Landrace	12.79	21	4
INCBN_02962	Indeterminate climbing	Southern Europe	Italy	Landrace	12.72	22	4
INCBN_01285	Indeterminate climbing	Western Europe	Austria	Landrace	12.68	23	4
INCBN_02059	Indeterminate climbing	Eastern Europe	Georgia	Landrace	12.63	24	4
INCBN_01222	Indeterminate climbing	Eastern Europe	Albania	Landrace	12.60	25	4
INCBN_02836	Indeterminate climbing	Southern Europe	Italy	Landrace	12.49	26	4
INCBN_00379	Determinate climbing	Southern Europe	Italy	Landrace	12.43	27	4
INCBN_01215	Indeterminate climbing	Eastern Europe	Albania	Landrace	12.41	28	4
INCBN_01807	Indeterminate climbing	Eastern Europe	Georgia	Landrace	12.33	29	4
INCBN_01434	Indeterminate climbing	Southern Europe	Croatia	Landrace	12.33	30	4

**Table 2:** Yield performance and stability ranking (ASV, WAAS) of R-core genotypes and their YSI classification.

R-Core Lines	Mean Yield	ASV	WAAS	Yield Rank	ASV Rank	YSI
INCBN_03223	425.35	2.25	2.25	7.00	1.00	8.00
INCBN_03300	479.42	23.00	23.00	4.00	5.00	9.00
INCBN_00143	400.89	16.45	16.45	10.00	3.00	13.00
INCBN_03273	513.24	109.44	109.44	2.00	12.00	14.00
INCBN_02842	243.30	11.26	11.26	13.00	2.00	15.00
INCBN_02066	414.51	37.26	37.26	9.00	7.00	16.00
INCBN_02002	422.57	68.04	68.04	8.00	8.00	16.00
INCBN_01323	509.35	117.39	117.39	3.00	13.00	16.00
INCBN_02957	384.45	24.45	24.45	11.00	6.00	17.00
INCBN_00433	427.08	149.09	149.09	6.00	14.00	20.00
INCBN_03286	514.05	232.77	232.77	1.00	19.00	20.00
INCBN_00474	127.49	21.60	21.60	17.00	4.00	21.00
INCBN_02821	475.97	199.71	199.71	5.00	17.00	22.00
INCBN_03046	238.65	70.73	70.73	14.00	9.00	23.00
INCBN_01359	305.55	92.90	92.90	12.00	11.00	23.00
INCBN_00413	46.99	90.89	90.89	19.00	10.00	29.00
INCBN_03229	205.60	168.66	168.66	15.00	15.00	30.00
INCBN_00111	126.95	194.75	194.75	18.00	16.00	34.00
INCBN_00444	128.10	205.57	205.57	16.00	18.00	34.00

ASV, AMMI Stability Value; WAAS, Weighted Average of Absolute Scores.

**Table 3:** Varimax-rotated factor loadings for the 20 quantitative traits in 2021 and 2022. Eigenvalues, variance, and cumulative variance for each factor are shown for each year.

	2021					2022					
Trait	FA1	FA2	FA3	FA4	FA5	FA1	FA2	FA3	FA4	FA5	FA6
Days to emergence	0.07	0.13	**0.82**	−0.13	−0.09	0.18	**0.79**	−0.07	−0.22	0.22	0.08
Emerged plants	0.03	−0.05	**−0.94**	0.06	−0.06	0.08	**−0.83**	−0.05	0.25	−0.08	0.21
Days to beginning of flowering	**−0.93**	−0.15	0.06	−0.15	0.11	**−0.83**	−0.05	0.15	−0.37	0.09	−0.14
Days to maximum flowering	**−0.98**	−0.03	0.02	−0.14	−0.02	**−0.91**	0.13	−0.04	−0.09	0.23	0.06
Days to end of flowering	**−0.48**	−0.22	0.23	−0.54	0.34	**−0.83**	0.12	0.09	0.38	0.09	−0.15
Days to pod formation	**−0.96**	−0.12	−0.07	−0.17	0.06	**−0.92**	−0.05	−0.04	0.02	0.10	−0.06
Full maturity	−0.43	0.06	0.18	**−0.80**	0.28	**−0.88**	0.08	−0.15	0.37	0.04	−0.11
Days to harvest	−0.43	0.06	0.18	**−0.80**	0.28	**−0.89**	0.05	−0.06	0.32	0.00	0.03
Number of plants with pods per plot	0.14	0.27	**−0.72**	0.22	−0.14	0.09	**−0.65**	−0.24	0.20	−0.05	0.45
Pod length	0.08	−0.17	0.08	0.23	**−0.86**	0.25	−0.42	0.05	−0.04	0.16	**0.76**
Pod width	−0.17	−0.13	0.02	**−0.78**	−0.17	−0.03	0.01	0.37	−0.07	0.11	**−0.88**
Weight of ten dry pods per plot	0.21	**0.64**	0.23	**−0.58**	−0.30	−0.20	−0.05	−0.08	0.07	**0.93**	−0.09
Number of seeds in ten dry pods per plot	0.26	**0.87**	0.20	−0.01	−0.05	0.36	0.12	−0.49	−0.47	0.54	0.05
Weight of total seeds in ten dry pods per plot	0.12	**0.71**	0.20	**−0.62**	−0.07	−0.38	0.25	0.03	0.16	**0.82**	0.09
1000 seed mass	−0.02	**0.26**	0.09	**−0.85**	0.05	−0.19	−0.41	−0.04	**0.78**	−0.14	0.10
Total number of seeds	0.07	**0.90**	−0.10	−0.09	0.14	−0.02	0.05	**−0.96**	−0.11	0.05	0.17
Total seed mass	0.08	**0.91**	−0.05	−0.14	0.06	−0.15	−0.19	**−0.88**	0.33	0.01	0.15
Useless seed mass	0.09	0.30	**−0.36**	−0.33	**−0.65**	−0.08	−0.06	−0.04	**0.87**	0.29	0.04
Plant canopy length	−0.17	0.77	−0.22	0.22	−0.18	0.17	**−0.57**	−0.47	−0.27	0.29	−0.38
Stem diameter	−0.32	−0.44	0.06	−0.38	−0.19	0.36	**−0.68**	0.05	−0.39	0.12	0.14
Eigenvalues	3.67	4.46	2.55	4.15	1.70	5.26	3.03	2.45	2.64	2.20	1.92
Variance (%)	18.37	22.30	12.76	20.76	8.49	26.31	15.14	12.23	13.2	11.02	9.61
Cumulative (%)	18.37	40.67	53.43	74.19	82.68	26.31	41.46	53.69	66.89	77.91	87.52

## Data Availability

Data are contained within the article.

## Supplementary Materials

The following supporting information can be downloaded at https://www.mdpi.com/article/10.3390/plants15081249/s1. Table S1: Descriptive statistics for the 20 quantitative traits evaluated in the R-core collection; Table S2: ANOVA results for quantitative traits across FAMD-HCPC clusters; Table S3: Complete Tukey HSD results for all quantitative traits across four grouping factors: Cluster_FAMD, Region, Growth Habit, and Biological Status; Table S4: Complete Selection Index (SI) dataset for all R-core lines; Table S5: Characteristics of the 19 R-core lines in HCPC Cluster 4 used in year-to-year analyses; Table S6: (A) List of *Phaseolus vulgaris* lines included in the phenotypic analyses (507 lines), (B) *Phaseolus vulgaris* lines excluded from the phenotypic analyses, (C) Subset of *Phaseolus vulgaris* lines evaluated across two growing seasons; Table S7: (A) Quantitative trait descriptors used in the INCREASE common-bean R-core phenotyping, (B) Qualitative trait descriptors used in the INCREASE common-bean R-core phenotyping; Table S8: (A) Quantitative traits included in the selection index (SI), their assigned weights, and breeding-based justification, (B) Qualitative traits included in the selection index (SI), their assigned weights, and breeding-based justification; Figure S1: Frequency distributions of 20 quantitative traits measured across 507 phenotyped lines. H′ indicates Shannon diversity index, and CV (%) indicates coefficient of variation; Figure S2: Frequency distribution of qualitative morphological and stress-related traits in the R-core common-bean lines. The Shannon diversity index (H′) is shown for each trait; Figure S3: Monthly temperature, precipitation, relative humidity, and solar radiation during the 2021 and 2022 growing seasons.

## Abbreviations

The following abbreviations are used in this manuscript:

AMMI	Additive Main Effects and Multiplicative Interaction
ASV	AMMI Stability Value
CV	Coefficient of Variation
DOI	Digital Object Identifier
FA	Factor Analysis
FAMD	Factor Analysis of Mixed Data
GGE	Genotype plus Genotype-by-Environment
HCPC	Hierarchical Clustering on Principal Components
IPCA	Interaction Principal Component Axis
MCA	Multiple Correspondence Analysis
MGIDI	Multi-trait Genotype–Ideotype Distance Index
PCA	Principal Component Analysis
SI	Selection Index
TSM	Total Seed Mass
WAAS	Weighted Average of Absolute Scores
YSI	Yield–Stability Index
