# Supplementary material for: Uncovering Phenotypic Variation in Common Bean (Phaseolus vulgaris L.): Insights from the INCREASE Project
Source: Plants (Basel). 2026 Apr 18;15(8):1249. doi: 10.3390/plants15081249 (PMC13120372; doi:10.3390/plants15081249)
Supplement: Supplementary file 1 [file plants-15-01249-s001.zip › plants-4226003-supplementary.pdf]

Uncovering Phenotypic Variation in Common Bean (*Phaseolus vulgaris* L.): Insights from the INCREASE Project

Hourieh Tavakoli Hasanaklou [1], Lovro Sinkovič [1], Roberto Papa [2], Elena Bitocchi [2], Elisa Bellucci [2], Peter Dolničar [1] and Barbara Pipan [1,*]

[1] Crop Science Department, Agricultural Institute of Slovenia, Hacquetova ulica 17, SI-1000 Ljubljana, Slovenia; hourieh.tavakolihasanaklou@kis.si (H.T.H.); lovro.sinkovic@kis.si (L.S.); peter.dolnicar@kis.si (P.D.)
[2] Department of Agricultural, Food and Environmental Sciences, Polytechnic University of Marche, via Brecce Bianche, 60131 Ancona, Italy; r.papa@staff.univpm.it (R.P.); e.bitocchi@univpm.it (E.B.); e.bellucci@univpm.it (E.B.)
* Correspondence: barbara.pipan@kis.si; Tel.: +386-1-280-52-73

Supplementary materials

Table S1. Descriptive statistics for 20 quantitative traits evaluated in the R-core collection.

Group of parameters	Descriptors	Mean	Min	Max	CV	SE
Emergence	Days to emergence	8.2	4	21	33.9	0.12
	Emerged plants	8.4	1	11	26	0.1
Flowering	Days to beginning of flowering	46.3	33	88	17.1	0.35
	Days to maximum flowering	50.6	38	101	17.6	0.4
	Days to the end of flowering	83.5	52	138	16.2	0.6
Pod development and physiological maturity	Days to pod formation	54.3	42	105	18.1	0.44
	Full maturity	106	70	146	17.3	0.82
	Days to harvest	111.3	57	171	14.9	0.74
	Number of plants with pods per plot	6.7	1	10	39	0.12
	Pod: length	12.3	1	23.5	21.5	0.12
Pod and seed production	Pod: width	7.5	0.5	18.7	66	0.22
	Weight of ten dry pods per plot	26.8	8.6	59.3	34	0.41
	Number of seeds in ten dry pods per plot	52.8	19	82	20.3	0.48
	Weight of total seeds in ten dry pods per plot	19.4	4	46	36.1	0.31
	1000 seed mass	33.3	8.9	66.7	33.8	0.5
	Total number of seeds	1064	27	3882	60.5	28.58
	Total seed mass	355.4	3.5	1083	60.8	9.6
	Useless seed mass	11	0	97	149.4	0.73
	Plant canopy length	214	35	2238	71.9	6.83
	Stem diameter	3.7	0.4	17	109.8	0.18

CV, coefficient of variation; SE, standard error.

Table S2. ANOVA results for quantitative traits across FAMD-HCPC clusters.

Group of parameters	Descriptors	Mean Square
Emergence	Days to emergence	324.8**
	Emerged plants	46.5**
Flowering	Days to beginning of flowering	386.5**
	Days to maximum flowering	573**
	Days to the end of flowering	247.9ns
Pod development and physiological maturity	Days to Pod formation	1203.3**
	Full maturity	20346**
	Days to harvest	3481**
	Number of plants with pods per plot	33.73**
	Pod: length	162.31**
	Pod: width	2742.9**
Pod and seed production	Weight of ten dry pods per plot	1257**
	Number of seeds in ten dry pods per plot	2372.4**
	Weight of total seeds in ten dry pods per plot	778.3**
	1000-seed mass	1106**
	Total number of seeds	1648449**
	Total seed mass	485799**
	Useless seed mass	4161**
	Plant canopy length	1793**
	Stem diameter	430131**

ns = not significant; ** = significant at $P < 0.001$.

Table S3. Complete Tukey HSD results for all quantitative traits across four grouping factors: Cluster_FAMD, Region, Growth Habit, and Biological Status.

	DE	EP	DBF	DMF	DEF	DPF	PCL	SD	FM	DH	NPP	PL	PW	WT	NST	WTS	W1000S	TNS	TSM	US
**Cluster_FAMD**																				
1	5.7^{b}	9.1^{ab}	43.8^{b}	49.5^{b}	84.9^{a}	57.4^{b}	110.1^{b}	8.1^{a}	85.6^{ab}	101.3^{ab}	6.5^{ab}	11.8^{ab}	1.0^{c}	22.5^{ab}	54.7^{ab}	16.1^{ab}	24.0^{ab}	1019.7^{ab}	242.3^{b}	10.3^{ab}
2	6.0^{b}	9.2^{a}	45.8^{b}	50.2^{b}	85.4^{a}	57.1^{b}	136.2^{b}	9.0^{a}	88.7^{ab}	104.3^{ab}	7.5^{a}	10.8^{ab}	1.1^{c}	22.5^{ab}	46.7^{ab}	16.0^{ab}	29.7^{ab}	921.7^{b}	276.3^{b}	19.2^{a}
3	7.0^{ab}	10.0^{a}	84.0^{a}	98.0^{a}	82.0^{a}	105.0^{a}	83.3^{b}	0.8^{b}	105.0^{ab}	105.0^{ab}	8.0^{a}	16.8^{a}	18.7^{a}	27.4^{ab}	34.0^{ab}	14.2^{ab}	37.5^{a}	40.0^{b}	15.0^{b}	0.0^{b}
4	9.4^{a}	7.9^{ab}	46.5^{b}	50.7^{b}	82.5^{a}	52.7^{b}	256.5^{a}	1.0^{b}	115.2^{a}	115.0^{a}	6.4^{ab}	13.1^{a}	10.8^{b}	29.1^{a}	55.8^{a}	21.2^{a}	35.3^{a}	1140.8^{a}	399.8^{a}	7.0^{b}
5	13.0^{a}	5.0^{ab}	50.0^{b}	52.0^{b}	75.0^{a}	54.0^{b}	175.0^{ab}	0.7^{b}	123.0^{a}	123.0^{a}	4.0^{ab}	16.5^{a}	14.7^{ab}	35.3^{a}	63.0^{a}	25.0^{a}	37.2^{a}	578.0^{b}	223.3^{b}	0.0^{b}
**Region**																				
America	7.1^{ab}	9.4^{ab}	44.0^{b}	48.0^{b}	86.3^{ab}	50.4^{b}	84.9^{b}	5.5^{ab}	100.3^{ab}	105.3^{ab}	6.7^{ab}	11.4^{ab}	5.0^{ab}	21.4^{ab}	51.9^{a}	15.5^{ab}	27.9^{ab}	862.4^{ab}	231.4^{ab}	15.3^{a}
Central Asia	6.0^{ab}	9.0^{ab}	80.0^{a}	82.0^{a}	112.0^{ab}	90.0^{a}	195.0^{ab}	8.0^{a}	112.0^{a}	124.0^{a}	9.0^{a}	9.0^{b}	1.0^{ab}	21.6^{ab}	47.0^{a}	16.6^{ab}	23.4^{ab}	1672.0^{ab}	406.8^{a}	10.0^{a}
Eastern Europe	8.2^{ab}	8.2^{ab}	45.5^{b}	50.2^{b}	83.2^{b}	53.9^{b}	207.6^{ab}	3.9^{ab}	107.2^{a}	112.2^{ab}	6.9^{ab}	12.4^{ab}	7.6^{ab}	28.6^{a}	51.8^{a}	21.0^{a}	36.3^{a}	1025.2^{ab}	377.5^{a}	10.8^{a}
North America	11.5^{a}	4.0^{ab}	44.0^{b}	49.0^{b}	77.5^{b}	50.0^{b}	106.7^{ab}	0.7^{ab}	95.5^{ab}	95.5^{ab}	4.5^{ab}	11.3^{ab}	9.1^{a}	17.5^{ab}	41.5^{a}	11.1^{b}	21.2^{ab}	612.5^{ab}	172.7^{ab}	4.3^{a}
Northern Europe	4.0^{ab}	10.0^{a}	38.0^{b}	42.0^{b}	66.0^{b}	47.0^{b}	38.3^{b}	7.0^{a}	73.0^{ab}	80.0^{ab}	10.0^{a}	13.0^{ab}	0.9^{ab}	26.8^{ab}	52.8^{a}	19.4^{ab}	22.5^{ab}	758.0^{ab}	178.9^{ab}	5.0^{a}
Southern Europe	8.8^{a}	8.1^{ab}	46.8^{b}	50.7^{b}	83.1^{b}	53.7^{b}	258.0^{a}	2.8^{ab}	109.4^{a}	112.5^{ab}	6.6^{ab}	12.8^{ab}	8.8^{a}	27.7^{a}	53.7^{a}	19.8^{a}	33.5^{a}	1143.9^{ab}	383.6^{a}	8.7^{a}
Unknown	7.3^{ab}	9.5^{ab}	52.9^{b}	58.6^{ab}	88.9^{ab}	64.7^{ab}	197.4^{ab}	6.6^{a}	99.9^{ab}	119.8^{a}	7.0^{ab}	13.4^{a}	5.2^{ab}	30.3^{a}	52.4^{a}	21.5^{a}	34.7^{a}	796.9^{ab}	256.8^{ab}	17.1^{a}
Central America	6.5^{ab}	9.6^{a}	46.6^{b}	51.5^{b}	81.6^{b}	56.6^{b}	105.2^{b}	5.2^{ab}	94.9^{ab}	101.9^{ab}	6.9^{ab}	10.4^{b}	4.2^{ab}	16.6^{b}	54.8^{a}	11.8^{b}	20.2^{b}	955.1^{ab}	203.8^{ab}	11.7^{a}
East Asia	7.1^{ab}	9.8^{a}	41.9^{b}	43.9^{b}	79.0^{b}	48.9^{b}	202.0^{ab}	4.4^{ab}	94.3^{ab}	98.1^{ab}	8.9^{a}	12.3^{ab}	7.4^{ab}	22.7^{ab}	60.6^{a}	15.8^{ab}	24.9^{ab}	1395.2^{ab}	346.9^{ab}	24.5^{a}
Middle East	7.6^{ab}	8.0^{ab}	46.3^{b}	49.6^{b}	82.3^{b}	55.6^{b}	230.1^{ab}	5.0^{ab}	99.1^{ab}	106.6^{ab}	6.9^{ab}	11.7^{ab}	5.4^{ab}	28.1^{a}	54.5^{a}	20.8^{a}	32.0^{ab}	1296.9^{ab}	423.5^{a}	16.3^{a}
Northern Africa	5.0^{ab}	9.0^{ab}	45.0^{b}	49.0^{b}	80.0^{b}	56.5^{b}	124.8^{ab}	9.0^{a}	85.5^{ab}	100.0^{ab}	6.5^{ab}	9.5^{b}	0.7^{ab}	17.2^{ab}	58.5^{a}	13.1^{ab}	20.4^{ab}	2301.5^{a}	503.2^{a}	30.0^{a}
South America	8.6^{ab}	8.3^{ab}	44.9^{b}	50.3^{b}	87.8^{ab}	54.6^{b}	211.0^{ab}	3.1^{ab}	111.8^{a}	116.2^{ab}	4.9^{ab}	12.7^{ab}	8.4^{ab}	24.7^{ab}	50.5^{a}	17.4^{ab}	31.9^{ab}	568.8^{b}	186.1^{ab}	4.4^{a}
Sub-Saharan Africa	6.0^{ab}	9.5^{ab}	60.0^{ab}	66.0^{ab}	114.5^{a}	71.5^{ab}	103.3^{b}	10.3^{a}	106.0^{ab}	140.0^{a}	3.5^{ab}	7.0^{b}	1.2^{ab}	22.8^{ab}	40.5^{a}	17.7^{ab}	33.3^{ab}	236.5^{b}	91.4^{ab}	12.1^{a}
Western Europe	8.3^{ab}	8.2^{ab}	45.6^{b}	49.4^{b}	82.4^{b}	52.5^{b}	214.1^{ab}	3.2^{ab}	104.4^{ab}	109.0^{ab}	6.6^{ab}	12.1^{ab}	7.1^{ab}	25.7^{ab}	52.0^{a}	18.7^{ab}	33.7^{a}	1216.7^{ab}	401.7^{a}	12.8^{a}
**Growth Habit**																				
Determinate bush	6.1^{b}	9.3^{a}	40.8^{b}	45.2^{b}	75.9^{c}	50.6^{b}	86.4^{c}	7.7^{a}	86.4^{b}	97.2^{c}	7.8^{a}	10.9^{c}	2.0^{c}	22.7^{b}	46.8^{b}	16.2^{b}	30.9^{ab}	825.1^{b}	257.7^{b}	19.0^{a}
Determinate climbing	8.7^{a}	8.5^{ab}	43.3^{b}	46.2^{b}	77.5^{c}	49.3^{b}	245.3^{ab}	3.2^{b}	104.8^{a}	107.4^{b}	6.9^{ab}	14.5^{a}	9.7^{a}	30.1^{a}	56.8^{a}	21.2^{a}	33.5^{ab}	996.7^{ab}	346.7^{ab}	10.1^{b}
Indeterminate bush	7.3^{ab}	8.9^{ab}	45.7^{ab}	50.8^{ab}	90.1^{ab}	54.1^{ab}	76.3^{c}	4.5^{b}	101.7^{a}	107.6^{ab}	6.5^{ab}	10.7^{c}	6.1^{b}	19.5^{b}	51.7^{ab}	14.1^{b}	26.2^{b}	885.7^{ab}	209.3^{b}	7.8^{b}
Indeterminate climbing	8.6^{a}	8.2^{b}	47.4^{a}	51.8^{a}	84.1^{b}	55.2^{a}	246.3^{a}	3.1^{b}	108.9^{a}	113.6^{ab}	6.7^{ab}	12.5^{b}	8.2^{ab}	28.2^{a}	53.4^{a}	20.5^{a}	34.6^{a}	1154.5^{a}	399.8^{a}	10.9^{b}
Indeterminate prostrate	7.8^{a}	8.2^{ab}	48.1^{a}	53.1^{a}	90.4^{a}	57.2^{a}	169.5^{b}	3.9^{b}	110.8^{a}	116.5^{a}	5.6^{b}	11.3^{c}	6.7^{b}	22.3^{b}	51.4^{ab}	16.4^{b}	29.9^{ab}	840.8^{b}	232.3^{b}	5.3^{b}
**Biological Status**																				
Breeding/research material	6.0^{b}	10.0^{a}	42.0^{a}	45.0^{a}	73.0^{b}	54.0^{ab}	94.0^{b}	9.0^{a}	80.0^{ab}	94.0^{a}	10.0^{a}	9.0^{b}	0.7^{ab}	26.8^{ab}	52.8^{ab}	19.4^{ab}	15.0^{a}	1676.0^{a}	265.5^{b}	11.1^{ab}
Cultivar	8.2^{ab}	8.0^{a}	43.6^{a}	47.4^{a}	76.7^{b}	49.6^{b}	189.9^{ab}	3.0^{ab}	101.7^{ab}	106.6^{a}	6.8^{a}	13.6^{a}	7.8^{a}	26.7^{ab}	55.9^{a}	18.8^{ab}	30.7^{a}	984.6^{ab}	305.6^{ab}	11.0^{ab}
Hybrid	7.0^{ab}	10.0^{a}	41.0^{a}	49.0^{a}	82.0^{b}	52.0^{b}	76.7^{b}	1.1^{ab}	110.0^{a}	110.0^{a}	3.0^{a}	11.0^{b}	10.4^{a}	20.1^{b}	53.0^{ab}	15.4^{b}	28.8^{a}	764.0^{ab}	216.1^{b}	0.0^{b}
Landrace	8.6^{a}	8.2^{a}	46.2^{a}	50.4^{a}	82.3^{b}	53.3^{b}	234.0^{a}	2.6^{ab}	109.7^{a}	112.3^{a}	6.7^{a}	12.6^{a}	8.8^{a}	27.7^{a}	54.3^{a}	20.1^{a}	34.0^{a}	1116.8^{a}	381.1^{a}	9.2^{b}
Unknown	6.7^{b}	8.9^{a}	47.4^{a}	52.2^{a}	89.5^{a}	59.1^{a}	146.6^{b}	8.1^{a}	93.2^{ab}	108.7^{a}	6.9^{a}	11.2^{b}	2.3^{ab}	23.8^{b}	46.2^{b}	16.9^{b}	31.3^{a}	879.1^{ab}	271.8^{b}	18.0^{a}

Table S4. Complete Selection Index (SI) dataset for all R-core liness.

Line	PH	GEO REGION	Country	Biological status	Selection Index	Rank SI	HCPC Cluster
INCBN 03268	indeterminate climbing	Eastern Europe	Slovakia	Landrace	15,06	1	4
INCBN 02799	indeterminate climbing	Southern Europe	Italy	Landrace	14,84	2	4
INCBN 01194	indeterminate climbing	Eastern Europe	Albania	Landrace	14,51	3	4
INCBN 03277	indeterminate climbing	Eastern Europe	Slovakia	Landrace	14,35	4	4
INCBN 00496	indeterminate climbing	Southern Europe	Spain	Landrace	14,30	5	4
INCBN 01417	indeterminate climbing	Southern Europe	Croatia	Landrace	14,17	6	4
INCBN 02052	indeterminate climbing	Eastern Europe	Georgia	Landrace	14,12	7	4
INCBN 00514	indeterminate climbing	Middle East	Turkey	Cultivar	14,04	8	4
INCBN 02759	indeterminate climbing	Southern Europe	Greece	Landrace	14,03	9	4
INCBN 01337	indeterminate climbing	Western Europe	Austria	Landrace	13,94	10	4
INCBN 01203	determinate climbing	Eastern Europe	Albania	Landrace	13,91	11	4
INCBN 01808	indeterminate climbing	Eastern Europe	Georgia	Landrace	13,80	12	4
INCBN 01423	indeterminate climbing	Southern Europe	Croatia	Landrace	13,33	13	4
INCBN 02933	indeterminate climbing	Southern Europe	Italy	Landrace	13,33	14	4
INCBN 03286	indeterminate climbing	Eastern Europe	Slovakia	Landrace	13,31	15	4
INCBN 01313	indeterminate climbing	Western Europe	Austria	Landrace	13,22	16	4
INCBN 01335	indeterminate climbing	Western Europe	Austria	Landrace	13,18	17	4
INCBN 01241	indeterminate climbing	Eastern Europe	Albania	Landrace	13,09	18	4
INCBN 01199	indeterminate climbing	Eastern Europe	Albania	Landrace	13,08	19	4
INCBN 00505	indeterminate climbing	Southern Europe	Spain	Landrace	12,91	20	4
INCBN 00489	indeterminate climbing	Eastern Europe	Bulgaria	Landrace	12,79	21	4
INCBN 02962	indeterminate climbing	Southern Europe	Italy	Landrace	12,72	22	4
INCBN 01285	indeterminate climbing	Western Europe	Austria	Landrace	12,68	23	4
INCBN 02059	indeterminate climbing	Eastern Europe	Georgia	Landrace	12,63	24	4
INCBN 01222	indeterminate climbing	Eastern Europe	Albania	Landrace	12,60	25	4
INCBN 02836	indeterminate climbing	Southern Europe	Italy	Landrace	12,49	26	4
INCBN 00379	determinate climbing	Southern Europe	Italy	Landrace	12,43	27	4
INCBN 01215	indeterminate climbing	Eastern Europe	Albania	Landrace	12,41	28	4
INCBN 01807	indeterminate climbing	Eastern Europe	Georgia	Landrace	12,33	29	4
INCBN 01434	indeterminate climbing	Southern Europe	Croatia	Landrace	12,33	30	4
INCBN 02838	indeterminate climbing	Southern Europe	Italy	Landrace	12,29	31	4
INCBN 00400	determinate climbing	Southern Europe	Spain	Landrace	12,25	32	4
INCBN 02830	indeterminate climbing	Southern Europe	Italy	Landrace	12,25	33	4
INCBN 01309	indeterminate climbing	Western Europe	Austria	Landrace	12,23	34	4
INCBN 00136	determinate climbing	Eastern Europe	Bulgaria	Landrace	12,17	35	4
INCBN 00833	indeterminate climbing	Southern Europe	Greece	Landrace	12,16	36	4
INCBN 00504	indeterminate climbing	Southern Europe	Spain	Landrace	12,13	37	4
INCBN 01432	indeterminate climbing	Southern Europe	Croatia	Landrace	12,13	38	4
INCBN 01306	indeterminate climbing	Western Europe	Austria	Landrace	12,10	39	4
INCBN 02017	indeterminate climbing	Eastern Europe	Georgia	Landrace	12,08	40	4
INCBN 00203	determinate climbing	Eastern Europe	Slovakia	Unknown	11,97	41	4
INCBN 02932	determinate climbing	Southern Europe	Italy	Landrace	11,93	42	4
INCBN 00430	indeterminate climbing	Southern Europe	Greece	Landrace	11,78	43	4
INCBN 00429	indeterminate climbing	Southern Europe	Greece	Landrace	11,68	44	4
INCBN 01426	indeterminate climbing	Southern Europe	Croatia	Landrace	11,63	45	4
INCBN 03267	indeterminate climbing	Eastern Europe	Slovakia	Landrace	11,62	46	4
INCBN 03273	indeterminate climbing	Eastern Europe	Slovakia	Landrace	11,60	47	4
INCBN 03015	indeterminate climbing	Southern Europe	Italy	Landrace	11,60	48	4
INCBN 02038	indeterminate climbing	Eastern Europe	Georgia	Landrace	11,51	49	4
INCBN 00206	indeterminate climbing	Southern Europe	Italy	Landrace	11,51	50	4
INCBN 03309	indeterminate climbing	Eastern Europe	Slovakia	Landrace	11,48	51	4
INCBN 02821	indeterminate climbing	Southern Europe	Italy	Landrace	11,48	52	4
INCBN 02036	indeterminate climbing	Eastern Europe	Georgia	Landrace	11,43	53	4
INCBN 03061	indeterminate climbing	Southern Europe	Italy	Landrace	11,43	54	4
INCBN 03207	indeterminate climbing	Eastern Europe	Slovakia	Landrace	11,42	55	4
INCBN 03512	determinate bush	Unknown	unknown	Cultivar	11,42	56	4
INCBN 03294	indeterminate climbing	Eastern Europe	Slovakia	Landrace	11,41	57	4
INCBN 01299	indeterminate climbing	Western Europe	Austria	Landrace	11,41	58	4
INCBN 02088	indeterminate climbing	Eastern Europe	Georgia	Landrace	11,41	59	4
INCBN 00055	indeterminate prostrate	America	Mexico	Landrace	11,37	60	4
INCBN 01284	determinate climbing	Western Europe	Austria	Landrace	11,35	61	4
INCBN 01780	indeterminate climbing	Eastern Europe	Georgia	Landrace	11,34	62	4
INCBN 03198	indeterminate climbing	Eastern Europe	Slovakia	Landrace	11,33	63	4
INCBN 01305	indeterminate climbing	Western Europe	Austria	Landrace	11,29	64	4
INCBN 00436	indeterminate climbing	Southern Europe	Greece	Landrace	11,28	65	4
INCBN 03210	indeterminate climbing	Eastern Europe	Slovakia	Landrace	11,27	66	4
INCBN 00145	indeterminate climbing	Eastern Europe	Czech Republic	Landrace	11,21	67	4
INCBN 01767	indeterminate prostrate	Eastern Europe	Georgia	Landrace	11,21	68	4
INCBN 03289	indeterminate climbing	Eastern Europe	Slovakia	Landrace	11,20	69	4
INCBN 00488	indeterminate climbing	Western Europe	Germany	Cultivar	11,18	70	4
INCBN 02953	indeterminate climbing	Southern Europe	Italy	Landrace	11,18	71	4
INCBN 00513	indeterminate climbing	Middle East	Turkey	Cultivar	11,16	72	4
INCBN 00445	indeterminate climbing	Southern Europe	Greece	Landrace	11,13	73	4
INCBN 01385	determinate climbing	East Asia	China	Landrace	11,09	74	4
INCBN 00135	indeterminate climbing	Eastern Europe	Albania	Landrace	11,08	75	4
INCBN 01889	indeterminate climbing	Eastern Europe	Georgia	Landrace	11,04	76	4
INCBN 02957	indeterminate climbing	Southern Europe	Italy	Landrace	11,04	77	4
INCBN 06736	indeterminate climbing	Eastern Europe	Romania	Unknown	11,04	78	2
INCBN 00848	indeterminate prostrate	Southern Europe	Italy	Landrace	10,98	79	4
INCBN 00494	determinate climbing	Southern Europe	Spain	Landrace	10,95	80	4
INCBN 01212	indeterminate climbing	Eastern Europe	Albania	Landrace	10,94	81	4
INCBN 09929	indeterminate climbing	Eastern Europe	Georgia	Unknown	10,92	82	2
INCBN 00874	indeterminate climbing	Southern Europe	Italy	Landrace	10,92	83	4

INCBN 03223	indeterminate climbing	Eastern Europe	Slovakia	Landrace	10,91	84	4
INCBN 02068	determinate climbing	Eastern Europe	Georgia	Landrace	10,83	85	4
INCBN 02960	indeterminate climbing	Southern Europe	Italy	Landrace	10,83	86	4
INCBN 00461	indeterminate climbing	Western Europe	Austria	Landrace	10,80	87	4
INCBN 03298	indeterminate climbing	Eastern Europe	Slovakia	Landrace	10,79	88	4
INCBN 00840	indeterminate climbing	Southern Europe	Greece	Landrace	10,78	89	4
INCBN 00502	indeterminate climbing	Southern Europe	Spain	Landrace	10,77	90	4
INCBN 00479	indeterminate climbing	Southern Europe	Spain	Landrace	10,77	91	4
INCBN 00459	indeterminate climbing	Western Europe	Austria	Landrace	10,74	92	4
INCBN 03047	indeterminate climbing	Southern Europe	Italy	Landrace	10,74	93	4
INCBN 02954	indeterminate climbing	Southern Europe	Italy	Landrace	10,70	94	4
INCBN 00975	indeterminate climbing	Southern Europe	Greece	Landrace	10,67	95	4
INCBN 00438	indeterminate climbing	Southern Europe	Greece	Landrace	10,65	96	4
INCBN 03300	indeterminate climbing	Eastern Europe	Slovakia	Landrace	10,60	97	4
INCBN 00293	determinate climbing	Central America	Guatemala	Unknown	10,58	98	4
INCBN 03248	indeterminate climbing	Eastern Europe	Slovakia	Landrace	10,56	99	4
INCBN 09985	indeterminate climbing	Unknown	unknown	Cultivar	10,54	100	4
INCBN 01740	indeterminate climbing	Eastern Europe	Georgia	Landrace	10,52	101	4
INCBN 03288	indeterminate climbing	Eastern Europe	Slovakia	Landrace	10,48	102	4
INCBN 01258	indeterminate climbing	Western Europe	Austria	Landrace	10,48	103	4
INCBN 01240	indeterminate climbing	Eastern Europe	Albania	Landrace	10,47	104	4
INCBN 01620	determinate climbing	Central America	Cuba	Landrace	10,46	105	4
INCBN 00478	indeterminate prostrate	Southern Europe	Spain	Landrace	10,43	106	4
INCBN 02002	indeterminate climbing	Eastern Europe	Georgia	Landrace	10,43	107	4
INCBN 01298	indeterminate climbing	Western Europe	Austria	Landrace	10,40	108	4
INCBN 00449	indeterminate climbing	Southern Europe	Italy	Landrace	10,39	109	4
INCBN 01310	indeterminate climbing	Western Europe	Austria	Landrace	10,39	110	4
INCBN 03085	indeterminate climbing	Eastern Europe	Poland	Landrace	10,37	111	4
INCBN 00469	indeterminate climbing	Southern Europe	Spain	Landrace	10,31	112	4
INCBN 02070	indeterminate climbing	Eastern Europe	Georgia	Landrace	10,28	113	4
INCBN 00516	indeterminate climbing	Eastern Europe	Bulgaria	Landrace	10,22	114	4
INCBN 03262	determinate bush	Eastern Europe	Slovakia	Landrace	10,20	115	4
INCBN 01764	indeterminate climbing	Eastern Europe	Georgia	Landrace	10,17	116	4
INCBN 02091	indeterminate climbing	Eastern Europe	Georgia	Landrace	10,17	117	4
INCBN 03201	indeterminate climbing	Eastern Europe	Slovakia	Landrace	10,13	118	4
INCBN 03305	indeterminate climbing	Eastern Europe	Slovakia	Landrace	10,11	119	4
INCBN 00121	indeterminate climbing	Southern Europe	Italy	Landrace	10,11	120	4
INCBN 00450	indeterminate climbing	Eastern Europe	Bulgaria	Landrace	10,10	121	2
INCBN 01237	indeterminate climbing	Eastern Europe	Albania	Landrace	10,08	122	4
INCBN 02895	indeterminate climbing	Southern Europe	Italy	Landrace	10,05	123	4
INCBN 00399	determinate bush	Eastern Europe	Georgia	Landrace	10,04	124	2
INCBN 00184	indeterminate climbing	Eastern Europe	Romania	Landrace	10,02	125	2
INCBN 02917	indeterminate climbing	Southern Europe	Italy	Landrace	10,00	126	4
INCBN 01289	indeterminate climbing	Western Europe	Austria	Landrace	10,00	127	4
INCBN 00443	indeterminate climbing	Southern Europe	Italy	Landrace	9,99	128	4
INCBN 01806	indeterminate climbing	Eastern Europe	Georgia	Landrace	9,96	129	4
INCBN 00113	determinate climbing	Eastern Europe	Georgia	Landrace	9,94	130	4
INCBN 00915	indeterminate climbing	Southern Europe	Italy	Landrace	9,93	131	4
INCBN 03301	indeterminate climbing	Eastern Europe	Slovakia	Landrace	9,89	132	4
INCBN 00464	indeterminate climbing	Eastern Europe	Georgia	Landrace	9,89	133	4
INCBN 00465	indeterminate climbing	Eastern Europe	Georgia	Landrace	9,88	134	4
INCBN 01409	indeterminate climbing	Southern Europe	Croatia	Landrace	9,87	135	4
INCBN 03264	indeterminate climbing	Eastern Europe	Slovakia	Landrace	9,86	136	4
INCBN 02888	indeterminate climbing	Southern Europe	Italy	Landrace	9,86	137	4
INCBN 03445	indeterminate climbing	Unknown	unknown	Cultivar	9,84	138	4
INCBN 02948	indeterminate climbing	Southern Europe	Italy	Landrace	9,82	139	4
INCBN 02855	indeterminate climbing	Southern Europe	Italy	Landrace	9,81	140	4
INCBN 00471	indeterminate climbing	Eastern Europe	Albania	Landrace	9,75	141	4
INCBN 03034	determinate climbing	Southern Europe	Italy	Landrace	9,74	142	4
INCBN 00297	indeterminate bush	America	Mexico	Unknown	9,68	143	4
INCBN 03261	indeterminate climbing	Eastern Europe	Slovakia	Landrace	9,67	144	4
INCBN 00435	determinate climbing	Middle East	Turkey	Landrace	9,66	145	4
INCBN 01441	indeterminate climbing	Southern Europe	Croatia	Landrace	9,66	146	4
INCBN 01804	indeterminate climbing	Eastern Europe	Georgia	Landrace	9,65	147	2
INCBN 00124	indeterminate climbing	Eastern Europe	Bulgaria	Landrace	9,64	148	4
INCBN 02011	indeterminate prostrate	Eastern Europe	Georgia	Landrace	9,63	149	4
INCBN 00509	indeterminate climbing	Southern Europe	Spain	Landrace	9,62	150	4
INCBN 01898	indeterminate climbing	Eastern Europe	Georgia	Landrace	9,62	151	4
INCBN 00096	indeterminate climbing	South America	Argentina	Landrace	9,60	152	4
INCBN 00051	determinate climbing	South America	Chile	Landrace	9,57	153	4
INCBN 01373	indeterminate climbing	East Asia	China	Landrace	9,55	154	4
INCBN 00372	determinate bush	Eastern Europe	Czech Republic	Unknown	9,55	155	2
INCBN 00130	determinate bush	Northern Europe	Sweden	Unknown	9,55	156	2
INCBN 00508	indeterminate climbing	Western Europe	Austria	Landrace	9,50	157	4
INCBN 00451	indeterminate prostrate	Southern Europe	Italy	Landrace	9,49	158	4
INCBN 01300	indeterminate bush	Western Europe	Austria	Landrace	9,45	159	4
INCBN 02746	indeterminate climbing	Southern Europe	Greece	Landrace	9,45	160	4
INCBN 00404	determinate bush	Southern Europe	Spain	Landrace	9,44	161	2
INCBN 02833	indeterminate climbing	Southern Europe	Italy	Landrace	9,43	162	4
INCBN 03241	indeterminate climbing	Eastern Europe	Slovakia	Landrace	9,41	163	4
INCBN 00183	determinate bush	Southern Europe	Portugal	Landrace	9,40	164	2
INCBN 09935	indeterminate climbing	Eastern Europe	Georgia	Unknown	9,40	165	2
INCBN 02952	indeterminate climbing	Southern Europe	Italy	Landrace	9,39	166	4
INCBN 00485	indeterminate climbing	Southern Europe	Croatia	Landrace	9,36	167	4
INCBN 03281	indeterminate climbing	Eastern Europe	Slovakia	Landrace	9,35	168	4
INCBN 02897	indeterminate climbing	Southern Europe	Italy	Landrace	9,35	169	4

INCBN 00120	determinate climbing	Southern Europe	Italy	Landrace	9,35	170	4
INCBN 01359	indeterminate climbing	Western Europe	Austria	Landrace	9,34	171	4
INCBN 00486	determinate climbing	Southern Europe	Croatia	Landrace	9,27	172	4
INCBN 02860	indeterminate climbing	Southern Europe	Italy	Landrace	9,26	173	4
INCBN 00432	determinate climbing	Southern Europe	Greece	Landrace	9,26	174	4
INCBN 03048	indeterminate climbing	Southern Europe	Italy	Landrace	9,25	175	4
INCBN 00473	determinate climbing	Eastern Europe	Albania	Landrace	9,24	176	4
INCBN 02863	indeterminate climbing	Southern Europe	Italy	Landrace	9,24	177	4
INCBN 00331	determinate bush	America	Mexico	Unknown	9,23	178	2
INCBN 03479	indeterminate climbing	Unknown	unknown	Cultivar	9,23	179	4
INCBN 02734	indeterminate climbing	Southern Europe	Greece	Landrace	9,22	180	4
INCBN 06476	determinate bush	Central America	Cuba	Unknown	9,21	181	2
INCBN 03213	indeterminate climbing	Eastern Europe	Slovakia	Landrace	9,21	182	4
INCBN 00824 B	indeterminate climbing	Southern Europe	Greece	Landrace	9,15	183	2
INCBN 09474	determinate bush	East Asia	China	Unknown	9,14	184	2
INCBN 00497	indeterminate climbing	Southern Europe	Spain	Landrace	9,14	185	4
INCBN 06809	indeterminate climbing	Northern Africa	Tunisia	Unknown	9,13	186	2
INCBN 03237	indeterminate climbing	Eastern Europe	Slovakia	Landrace	9,12	187	4
INCBN 03292	indeterminate climbing	Eastern Europe	Slovakia	Landrace	9,11	188	4
INCBN 00924	indeterminate climbing	Eastern Europe	Albania	Landrace	9,10	189	4
INCBN 00202	determinate climbing	Southern Europe	Portugal	Landrace	9,08	190	4
INCBN 07388	determinate bush	Middle East	Turkey	Unknown	9,04	191	2
INCBN 00483	indeterminate climbing	Southern Europe	Croatia	Landrace	9,03	192	4
INCBN 02856	indeterminate climbing	Southern Europe	Italy	Landrace	8,99	193	4
INCBN 00491	indeterminate climbing	Eastern Europe	Bulgaria	Landrace	8,96	194	4
INCBN 02058	indeterminate climbing	Eastern Europe	Georgia	Landrace	8,94	195	4
INCBN 02749	indeterminate climbing	Southern Europe	Greece	Landrace	8,94	196	4
INCBN 00974	indeterminate climbing	Southern Europe	Greece	Landrace	8,92	197	4
INCBN 03435	indeterminate climbing	Unknown	unknown	Landrace	8,92	198	4
INCBN 00439	indeterminate climbing	Middle East	Turkey	Landrace	8,89	199	4
INCBN 01323	indeterminate prostrate	Western Europe	Austria	Landrace	8,89	200	4
INCBN 10381	indeterminate climbing	Eastern Europe	Romania	Unknown	8,89	201	2
INCBN 10028	determinate bush	Unknown	unknown	Cultivar	8,86	202	2
INCBN 03263	indeterminate climbing	Eastern Europe	Slovakia	Landrace	8,86	203	4
INCBN 00384	determinate bush	Southern Europe	Greece	Landrace	8,83	204	2
INCBN 03509	indeterminate prostrate	Unknown	unknown	breeding/research material	8,82	205	1
INCBN 01933	indeterminate climbing	Eastern Europe	Georgia	Landrace	8,82	206	4
INCBN 03257	indeterminate climbing	Eastern Europe	Slovakia	Landrace	8,81	207	4
INCBN 00463	indeterminate climbing	Western Europe	Austria	Landrace	8,80	208	4
INCBN 01609	indeterminate bush	Central America	Cuba	Landrace	8,75	209	4
INCBN 00393	indeterminate bush	Eastern Europe	Georgia	Landrace	8,75	210	4
INCBN 00127	indeterminate climbing	Southern Europe	Slovenia	Unknown	8,73	211	4
INCBN 01376	indeterminate bush	East Asia	China	Landrace	8,73	212	4
INCBN 00395	determinate climbing	Eastern Europe	Georgia	Landrace	8,72	213	4
INCBN 00341	determinate bush	America	Mexico	Unknown	8,72	214	2
INCBN 03291	determinate climbing	Eastern Europe	Slovakia	Landrace	8,71	215	4
INCBN 03053	indeterminate climbing	Southern Europe	Italy	Landrace	8,70	216	4
INCBN 00433	indeterminate climbing	Southern Europe	Greece	Landrace	8,70	217	4
INCBN 00460	indeterminate climbing	Western Europe	Austria	Landrace	8,70	218	4
INCBN 00172	determinate climbing	Eastern Europe	Romania	Landrace	8,67	219	5
INCBN 00506	determinate climbing	Southern Europe	Spain	Landrace	8,66	220	2
INCBN 00495	indeterminate climbing	Southern Europe	Spain	Landrace	8,64	221	4
INCBN 00019	determinate climbing	South America	Chile	Cultivar	8,64	222	4
INCBN 03028	indeterminate climbing	Southern Europe	Italy	Landrace	8,64	223	4
INCBN 00180	indeterminate prostrate	Southern Europe	Italy	Landrace	8,61	224	4
INCBN 03222	indeterminate climbing	Eastern Europe	Slovakia	Landrace	8,61	225	4
INCBN 00480	indeterminate climbing	Southern Europe	Portugal	Landrace	8,60	226	4
INCBN 03366	determinate climbing	Unknown	unknown	Cultivar	8,59	227	2
INCBN 00344	indeterminate bush	Unknown	unknown	Unknown	8,58	228	2
INCBN 03260	indeterminate climbing	Eastern Europe	Slovakia	Landrace	8,57	229	4
INCBN 02761	indeterminate climbing	Southern Europe	Greece	Landrace	8,56	230	2
INCBN 03059	indeterminate climbing	Southern Europe	Italy	Landrace	8,56	231	4
INCBN 03383	determinate climbing	Unknown	unknown	Landrace	8,56	232	2
INCBN 02066	indeterminate climbing	Eastern Europe	Georgia	Landrace	8,54	233	4
INCBN 00462	indeterminate climbing	Western Europe	Austria	Landrace	8,54	234	4
INCBN 00116	determinate climbing	Southern Europe	Portugal	Landrace	8,53	235	4
INCBN 00174	determinate bush	Western Europe	Germany	Cultivar	8,49	236	1
INCBN 01943	indeterminate climbing	Eastern Europe	Georgia	Landrace	8,49	237	4
INCBN 00003	determinate bush	South America	Brazil	Landrace	8,48	238	4
INCBN 00431	indeterminate prostrate	Southern Europe	Greece	Landrace	8,45	239	4
INCBN 00108	determinate bush	Western Europe	Germany	Landrace	8,43	240	1
INCBN 06565	indeterminate climbing	Eastern Europe	Hungary	Unknown	8,43	241	2
INCBN 02823	indeterminate climbing	Southern Europe	Italy	Landrace	8,42	242	2
INCBN 03163	indeterminate climbing	Eastern Europe	Slovakia	Landrace	8,41	243	2
INCBN 02857	indeterminate climbing	Southern Europe	Italy	Landrace	8,37	244	4
INCBN 03536	indeterminate climbing	North America	USA	Cultivar	8,37	245	4
INCBN 00159	indeterminate climbing	Southern Europe	Greece	Landrace	8,36	246	4
INCBN 01328	indeterminate climbing	Western Europe	Austria	Landrace	8,35	247	4
INCBN 00920	indeterminate climbing	Eastern Europe	Albania	Landrace	8,35	248	4
INCBN 00194	determinate bush	Eastern Europe	Bulgaria	Landrace	8,34	249	2
INCBN 00470	indeterminate climbing	Southern Europe	Spain	Landrace	8,34	250	4
INCBN 02042	indeterminate bush	Eastern Europe	Georgia	Landrace	8,34	251	4
INCBN 01374	indeterminate climbing	East Asia	China	Landrace	8,34	252	4
INCBN 00824_A	indeterminate climbing	Southern Europe	Greece	Landrace	8,33	253	2
INCBN 03275	indeterminate prostrate	Eastern Europe	Slovakia	Landrace	8,33	254	4
INCBN 03299	indeterminate climbing	Eastern Europe	Slovakia	Landrace	8,32	255	4

INCBN 01914	indeterminate climbing	Eastern Europe	Georgia	Landrace	8,31	256	4
INCBN 00441	indeterminate climbing	Eastern Europe	Poland	Landrace	8,31	257	4
INCBN 02031	indeterminate climbing	Eastern Europe	Georgia	Landrace	8,30	258	4
INCBN 00327	determinate bush	South America	Ecuador	Unknown	8,30	259	2
INCBN 00187	determinate bush	Eastern Europe	Hungary	Landrace	8,30	260	2
INCBN 02828	indeterminate climbing	Southern Europe	Italy	Landrace	8,29	261	4
INCBN 01297	indeterminate climbing	Western Europe	Austria	Landrace	8,28	262	4
INCBN 00048	indeterminate climbing	South America	Peru	Landrace	8,22	263	4
INCBN 03229	indeterminate prostrate	Eastern Europe	Slovakia	Landrace	8,21	264	4
INCBN 00389	determinate bush	Eastern Europe	Bulgaria	Landrace	8,20	265	2
INCBN 01288	indeterminate prostrate	Western Europe	Austria	Landrace	8,19	266	4
INCBN 00507	indeterminate climbing	Southern Europe	Spain	Landrace	8,17	267	4
INCBN 02736	indeterminate climbing	Southern Europe	Greece	Landrace	8,17	268	4
INCBN 02760	indeterminate prostrate	Southern Europe	Greece	Landrace	8,16	269	1
INCBN 01868	indeterminate climbing	Eastern Europe	Georgia	Landrace	8,14	270	2
INCBN 03023	determinate bush	Southern Europe	Italy	Landrace	8,14	271	2
INCBN 10382	indeterminate climbing	Western Europe	France	Unknown	8,14	272	2
INCBN 07540	indeterminate climbing	Middle East	Turkey	Unknown	8,14	273	2
INCBN 02851	determinate bush	Southern Europe	Italy	Landrace	8,13	274	4
INCBN 00137	indeterminate climbing	Eastern Europe	Romania	Landrace	8,13	275	4
INCBN 00247	determinate bush	America	Mexico	Unknown	8,13	276	2
INCBN 01268	indeterminate climbing	Western Europe	Austria	Landrace	8,12	277	4
INCBN 00284	indeterminate bush	America	Mexico	Unknown	8,11	278	4
INCBN 01526	indeterminate climbing	Central America	Cuba	Landrace	8,11	279	4
INCBN 03220	determinate climbing	Eastern Europe	Slovakia	Landrace	8,11	280	2
INCBN 00020	indeterminate climbing	South America	Colombia	Cultivar	8,08	281	4
INCBN 02972	determinate climbing	Southern Europe	Italy	Landrace	8,07	282	4
INCBN 02737	indeterminate climbing	Southern Europe	Greece	Landrace	8,06	283	4
INCBN 06884	indeterminate climbing	Eastern Europe	Ukraine	Unknown	8,04	284	2
INCBN 00134	determinate climbing	Southern Europe	Italy	Unknown	8,04	285	4
INCBN 00128	indeterminate climbing	Eastern Europe	Yugoslavia	Landrace	8,03	286	4
INCBN 02054	indeterminate climbing	Eastern Europe	Georgia	Landrace	8,02	287	4
INCBN 02027	indeterminate climbing	Eastern Europe	Georgia	Landrace	8,01	288	4
INCBN 01992_B	indeterminate climbing	Eastern Europe	Georgia	Landrace	7,99	289	2
INCBN 02980	determinate climbing	Southern Europe	Italy	Landrace	7,97	290	4
INCBN 02894	indeterminate climbing	Southern Europe	Italy	Landrace	7,93	291	4
INCBN 00858	indeterminate climbing	Southern Europe	Italy	Landrace	7,93	292	2
INCBN 01654	indeterminate prostrate	Central America	Cuba	Landrace	7,92	293	2
INCBN 06473	indeterminate bush	Central America	Cuba	Unknown	7,91	294	2
INCBN 00950	indeterminate climbing	Southern Europe	Italy	Landrace	7,90	295	2
INCBN 01998	indeterminate climbing	Eastern Europe	Georgia	Landrace	7,90	296	4
INCBN 09634	indeterminate climbing	Western Europe	Austria	Unknown	7,89	297	2
INCBN 00112	determinate bush	Eastern Europe	Czech Republic	Landrace	7,85	298	2
INCBN 06881	indeterminate climbing	Eastern Europe	Ukraine	Unknown	7,83	299	2
INCBN 00152	determinate climbing	Southern Europe	Italy	Landrace	7,82	300	2
INCBN 00472	indeterminate climbing	Eastern Europe	Albania	Landrace	7,82	301	4
INCBN 01369	indeterminate climbing	East Asia	China	Landrace	7,81	302	4
INCBN 02859	indeterminate climbing	Southern Europe	Italy	Landrace	7,81	303	4
INCBN 07070	determinate climbing	Southern Europe	Spain	Unknown	7,81	304	2
INCBN 02801	indeterminate bush	Southern Europe	Italy	Landrace	7,80	305	2
INCBN 02024	indeterminate bush	Eastern Europe	Georgia	Landrace	7,80	306	4
INCBN 02864	indeterminate climbing	Southern Europe	Italy	Landrace	7,73	307	4
INCBN 00123	determinate climbing	Eastern Europe	Bulgaria	Landrace	7,72	308	4
INCBN 02842	indeterminate climbing	Southern Europe	Italy	Landrace	7,71	309	4
INCBN 00066	indeterminate bush	South America	Chile	Landrace	7,71	310	4
INCBN 00651	indeterminate prostrate	Southern Europe	Italy	Landrace	7,67	311	2
INCBN 00440	indeterminate climbing	Southern Europe	Greece	Landrace	7,67	312	4
INCBN 02822	indeterminate climbing	Southern Europe	Italy	Landrace	7,66	313	4
INCBN 01992_A	indeterminate climbing	Eastern Europe	Georgia	Landrace	7,65	314	2
INCBN 09971	indeterminate climbing	Eastern Europe	Georgia	Unknown	7,64	315	2
INCBN 03228	indeterminate climbing	Eastern Europe	Slovakia	Landrace	7,64	316	1
INCBN 00295	determinate bush	America	Mexico	Unknown	7,63	317	2
INCBN 00398	indeterminate prostrate	Eastern Europe	Georgia	Landrace	7,63	318	2
INCBN 03190_B	indeterminate climbing	Eastern Europe	Slovakia	Landrace	7,62	319	2
INCBN 03190_A	indeterminate climbing	Eastern Europe	Slovakia	Landrace	7,59	320	2
INCBN 00072	indeterminate bush	Central America	Costa Rica	Landrace	7,59	321	4
INCBN 00375	determinate bush	Eastern Europe	Slovakia	Unknown	7,59	322	2
INCBN 06455	indeterminate bush	Central America	Cuba	Unknown	7,58	323	2
INCBN 00323	indeterminate prostrate	South America	Brazil	Unknown	7,56	324	4
INCBN 00510	indeterminate prostrate	Southern Europe	Spain	Landrace	7,56	325	4
INCBN 00425	determinate bush	Eastern Europe	USSR	Cultivar	7,54	326	2
INCBN 03385	indeterminate prostrate	Unknown	unknown	Hybrid	7,52	327	4
INCBN 00302	determinate bush	America	Mexico	Unknown	7,50	328	2
INCBN 06385	indeterminate climbing	Eastern Europe	Albania	Unknown	7,50	329	2
INCBN 07222	determinate bush	Middle East	Syria	Unknown	7,49	330	2
INCBN 00394	determinate bush	Eastern Europe	Georgia	Landrace	7,49	331	2
INCBN 00114	indeterminate climbing	Eastern Europe	Georgia	Landrace	7,39	332	4
INCBN 01286	indeterminate bush	Western Europe	Austria	Landrace	7,38	333	4
INCBN 00698	indeterminate climbing	Eastern Europe	Georgia	Landrace	7,36	334	2
INCBN 07161	determinate bush	Southern Europe	Spain	Unknown	7,35	335	2
INCBN 00011	indeterminate climbing	South America	Peru	Landrace	7,35	336	4
INCBN 01333	indeterminate climbing	Western Europe	Austria	Landrace	7,32	337	4
INCBN 00895	indeterminate climbing	Western Europe	Austria	Landrace	7,31	338	4
INCBN 10370	indeterminate climbing	Eastern Europe	Romania	Unknown	7,31	339	2
INCBN 01779	indeterminate climbing	Eastern Europe	Georgia	Landrace	7,30	340	2
INCBN 02110	determinate bush	Eastern Europe	Georgia	Landrace	7,29	341	4

INCBN 00512	indeterminate climbing	Middle East	Iran	Unknown	7,29	342	4
INCBN 00520	indeterminate climbing	Eastern Europe	Slovakia	Landrace	7,24	343	4
INCBN 06659	indeterminate climbing	Central Asia	Kazakhstan	Unknown	7,24	344	2
INCBN 01377	determinate climbing	East Asia	China	Landrace	7,23	345	4
INCBN 00381	determinate climbing	Southern Europe	Italy	Landrace	7,22	346	4
INCBN 03194	indeterminate prostrate	Eastern Europe	Slovakia	Landrace	7,21	347	4
INCBN 00041	determinate bush	South America	Argentina	Landrace	7,21	348	2
INCBN 00346	determinate bush	Unknown	unknown	Unknown	7,20	349	2
INCBN 00143	indeterminate prostrate	Western Europe	Austria	Landrace	7,19	350	4
INCBN 03196	indeterminate climbing	Eastern Europe	Slovakia	Landrace	7,19	351	2
INCBN 00057	indeterminate prostrate	America	Mexico	Landrace	7,18	352	4
INCBN 00474	indeterminate climbing	Eastern Europe	Albania	Landrace	7,17	353	4
INCBN 00446	indeterminate climbing	Southern Europe	Italy	Landrace	7,14	354	4
INCBN 00148	indeterminate climbing	Eastern Europe	Czech Republic	Landrace	7,14	355	4
INCBN 00231	determinate bush	Central America	Guatemala	Unknown	7,10	356	2
INCBN 02731	indeterminate climbing	Western Europe	Germany	Cultivar	7,09	357	4
INCBN 01629	indeterminate prostrate	Central America	Cuba	Landrace	7,08	358	2
INCBN 09635	indeterminate climbing	Western Europe	Austria	Unknown	7,04	359	2
INCBN 02849	indeterminate climbing	Southern Europe	Italy	Landrace	7,03	360	2
INCBN 06452	indeterminate climbing	Central America	Cuba	Unknown	7,02	361	2
INCBN 02891	indeterminate climbing	Southern Europe	Italy	Landrace	7,01	362	4
INCBN_10641	indeterminate climbing	Unknown	unknown	Unknown	7,00	363	2
INCBN 09250	indeterminate climbing	East Asia	North Korea	Unknown	7,00	364	2
INCBN 00969	indeterminate prostrate	Eastern Europe	Bulgaria	Landrace	6,99	365	2
INCBN 10385	indeterminate climbing	Western Europe	France	Unknown	6,99	366	2
INCBN 00291	determinate bush	Central America	Guatemala	Unknown	6,98	367	2
INCBN 01881	indeterminate climbing	Eastern Europe	Georgia	Landrace	6,98	368	4
INCBN 00158	indeterminate climbing	Southern Europe	Greece	Landrace	6,96	369	4
INCBN 00826	indeterminate climbing	Southern Europe	Greece	Landrace	6,96	370	4
INCBN 01524	indeterminate bush	Central America	Cuba	Landrace	6,94	371	2
INCBN 00380	indeterminate climbing	Southern Europe	Italy	Landrace	6,92	372	4
INCBN 01202	indeterminate climbing	Eastern Europe	Albania	Landrace	6,92	373	4
INCBN 03101	indeterminate climbing	Eastern Europe	Slovakia	Landrace	6,91	374	2
INCBN 06563	indeterminate climbing	Eastern Europe	Hungary	Unknown	6,91	375	2
INCBN 00075	indeterminate climbing	Central America	Costa Rica	Landrace	6,91	376	4
INCBN 01658	indeterminate prostrate	Central America	Cuba	Landrace	6,90	377	2
INCBN 06892	indeterminate prostrate	Eastern Europe	Ukraine	Unknown	6,80	378	2
INCBN 00146	indeterminate climbing	Eastern Europe	Czech Republic	Landrace	6,80	379	1
INCBN 10383	indeterminate climbing	Western Europe	France	Unknown	6,78	380	2
INCBN 06440	indeterminate climbing	Eastern Europe	Bulgaria	Unknown	6,72	381	2
INCBN 02853	indeterminate climbing	Southern Europe	Italy	Landrace	6,72	382	4
INCBN 00225	indeterminate climbing	America	Mexico	Unknown	6,70	383	4
INCBN 00448	indeterminate climbing	Southern Europe	Italy	Landrace	6,70	384	4
INCBN 10378	indeterminate climbing	Eastern Europe	Romania	Unknown	6,64	385	2
INCBN 00875	indeterminate climbing	Southern Europe	Italy	Landrace	6,63	386	4
INCBN 00444	indeterminate climbing	Southern Europe	Italy	Landrace	6,63	387	4
INCBN 00837	indeterminate climbing	Southern Europe	Greece	Landrace	6,63	388	2
INCBN 00089	indeterminate prostrate	South America	Venenzuela	Landrace	6,60	389	4
INCBN 06690	indeterminate climbing	Eastern Europe	Poland	Unknown	6,56	390	2
INCBN 01553	indeterminate prostrate	Central America	Cuba	Landrace	6,55	391	4
INCBN 03046	indeterminate climbing	Southern Europe	Italy	Landrace	6,55	392	4
INCBN 02872	indeterminate prostrate	Southern Europe	Italy	Landrace	6,55	393	2
INCBN 03188	indeterminate climbing	Eastern Europe	Slovakia	Landrace	6,53	394	2
INCBN 00842	indeterminate climbing	Southern Europe	Greece	Landrace	6,50	395	2
INCBN 10368	indeterminate climbing	Eastern Europe	Romania	Unknown	6,48	396	2
INCBN 10386	indeterminate climbing	Western Europe	France	Unknown	6,46	397	2
INCBN 00260	determinate bush	America	Mexico	Unknown	6,41	398	2
INCBN 10375	indeterminate climbing	Eastern Europe	Romania	Unknown	6,37	399	2
INCBN 06503	determinate bush	Western Europe	France	Unknown	6,33	400	2
INCBN 00149	indeterminate climbing	Eastern Europe	Czech Republic	Landrace	6,31	401	4
INCBN 00201	indeterminate prostrate	Southern Europe	Greece	Landrace	6,31	402	4
INCBN 01496	indeterminate prostrate	Central America	Cuba	Landrace	6,30	403	2
INCBN 00256	indeterminate bush	Central America	Guatemala	Unknown	6,30	404	4
INCBN 08834	indeterminate climbing	East Asia	China	Unknown	6,29	405	2
INCBN 02882	indeterminate climbing	Southern Europe	Italy	Landrace	6,27	406	2
INCBN 00873	indeterminate climbing	Southern Europe	Italy	Landrace	6,26	407	2
INCBN 06458	indeterminate prostrate	Central America	Cuba	Unknown	6,22	408	2
INCBN 02994	indeterminate climbing	Southern Europe	Italy	Landrace	6,20	409	4
INCBN 00198	indeterminate climbing	Eastern Europe	Czechoslovakia	Landrace	6,19	410	4
INCBN 10380	indeterminate climbing	Eastern Europe	Romania	Unknown	6,18	411	2
INCBN 09693	indeterminate climbing	Eastern Europe	Georgia	Unknown	6,16	412	2
INCBN 06451	indeterminate bush	Central America	Cuba	Unknown	6,13	413	1
INCBN 06779	indeterminate climbing	Eastern Europe	Slovakia	Unknown	6,12	414	2
INCBN 00378	determinate climbing	Southern Europe	Italy	Landrace	6,05	415	4
INCBN_10634	indeterminate climbing	Unknown	unknown	Unknown	6,05	416	2
INCBN 09226	indeterminate climbing	Western Europe	Germany	Unknown	6,03	417	1
INCBN 06708	indeterminate climbing	Eastern Europe	Romania	Unknown	6,03	418	2
INCBN 06631	indeterminate climbing	Middle East	Iraq	Unknown	6,00	419	2
INCBN 06858	indeterminate climbing	Middle East	Turkey	Unknown	5,97	420	2
INCBN 10026	indeterminate climbing	Unknown	unknown	Cultivar	5,96	421	4
INCBN 00851	indeterminate climbing	Southern Europe	Greece	Landrace	5,89	422	2
INCBN 00354	indeterminate climbing	Unknown	unknown	Unknown	5,86	423	3
INCBN 09758	indeterminate prostrate	Middle East	Iran	Unknown	5,85	424	2
INCBN 03426	determinate climbing	Unknown	unknown	Cultivar	5,83	425	2
INCBN 06704	indeterminate climbing	Eastern Europe	Poland	Unknown	5,81	426	2
INCBN 02098	indeterminate climbing	Eastern Europe	Georgia	Landrace	5,81	427	4

INCBN 02771_B	indeterminate climbing	Southern Europe	Greece	Landrace	5,80	428	1
INCBN 07147	determinate bush	Southern Europe	Spain	Unknown	5,79	429	2
INCBN 01783	indeterminate climbing	Eastern Europe	Georgia	Landrace	5,79	430	2
INCBN 00519	indeterminate prostrate	Southern Europe	Greece	Landrace	5,77	431	4
INCBN 02012	indeterminate climbing	Eastern Europe	Georgia	Landrace	5,76	432	4
INCBN 00147	indeterminate prostrate	Eastern Europe	Czech Republic	Landrace	5,75	433	4
INCBN 01579	indeterminate climbing	Central America	Cuba	Landrace	5,70	434	2
INCBN 10361	indeterminate climbing	Eastern Europe	Romania	Unknown	5,69	435	4
INCBN 06666	indeterminate climbing	Northern Africa	Morocco	Unknown	5,66	436	2
INCBN 00188	determinate bush	Western Europe	Germany	Landrace	5,65	437	2
INCBN 08912	indeterminate bush	Eastern Europe	Hungary	Unknown	5,62	438	2
INCBN 00139	indeterminate bush	Western Europe	France	Landrace	5,62	439	1
INCBN_10645	indeterminate climbing	Unknown	unknown	Unknown	5,61	440	2
INCBN 00081	determinate climbing	Central America	El Salvador	Landrace	5,61	441	4
INCBN 06540	determinate bush	Western Europe	France	Unknown	5,59	442	2
INCBN 09154	indeterminate climbing	Western Europe	Austria	Unknown	5,53	443	2
INCBN 00482	determinate bush	Southern Europe	Croatia	Landrace	5,51	444	2
INCBN 00484	indeterminate climbing	Southern Europe	Croatia	Landrace	5,48	445	4
INCBN 06604	indeterminate climbing	Eastern Europe	Hungary	Unknown	5,46	446	2
INCBN 00090	indeterminate prostrate	America	Mexico	Cultivar	5,46	447	4
INCBN 08923	determinate bush	Eastern Europe	Poland	Unknown	5,42	448	2
INCBN 00457	indeterminate climbing	Eastern Europe	Poland	Landrace	5,41	449	4
INCBN 02410	determinate bush	Western Europe	Germany	Cultivar	5,41	450	2
INCBN 00157	indeterminate climbing	Eastern Europe	Albania	Landrace	5,36	451	2
INCBN 02771_A	indeterminate climbing	Southern Europe	Greece	Landrace	5,35	452	2
INCBN 02832	indeterminate climbing	Southern Europe	Italy	Landrace	5,33	453	2
INCBN 00817	indeterminate climbing	Southern Europe	Greece	Landrace	5,33	454	4
INCBN 00515	indeterminate climbing	Eastern Europe	Hungary	Cultivar	5,32	455	4
INCBN 02762	indeterminate climbing	Southern Europe	Greece	Landrace	5,29	456	4
INCBN 01530	indeterminate climbing	Central America	Cuba	Landrace	5,19	457	4
INCBN_10635	indeterminate climbing	Unknown	unknown	Unknown	5,16	458	2
INCBN 00437	indeterminate prostrate	Southern Europe	Greece	Landrace	5,14	459	4
INCBN 10384	indeterminate climbing	Western Europe	France	Unknown	5,10	460	2
INCBN 00173	determinate climbing	Western Europe	Austria	Landrace	5,08	461	4
INCBN 02009	indeterminate bush	Eastern Europe	Georgia	Landrace	5,04	462	4
INCBN 02841	indeterminate prostrate	Southern Europe	Italy	Landrace	5,00	463	2
INCBN 02765	indeterminate prostrate	Southern Europe	Greece	Landrace	4,92	464	4
INCBN 00319	determinate bush	South America	Colombia	Unknown	4,91	465	2
INCBN 02226	indeterminate climbing	Eastern Europe	Georgia	Landrace	4,84	466	2
INCBN_10642	indeterminate climbing	Unknown	unknown	Unknown	4,81	467	2
INCBN 00230	determinate bush	South America	Peru	Unknown	4,72	468	2
INCBN 02764	determinate climbing	Southern Europe	Greece	Landrace	4,70	469	4
INCBN 00054	indeterminate climbing	America	Mexico	Landrace	4,65	470	4
INCBN 02240	determinate bush	Eastern Europe	Georgia	Landrace	4,60	471	2
INCBN 03055	indeterminate bush	Southern Europe	Italy	Landrace	4,59	472	2
INCBN 10371	indeterminate climbing	Eastern Europe	Romania	Unknown	4,56	473	2
INCBN 02950	indeterminate climbing	Southern Europe	Italy	Landrace	4,51	474	4
INCBN 00477	indeterminate climbing	Southern Europe	Spain	Landrace	4,47	475	4
INCBN 09695	indeterminate climbing	Eastern Europe	Georgia	Unknown	4,47	476	2
INCBN 00052	indeterminate prostrate	South America	Ecuador	Landrace	4,42	477	4
INCBN 06547	indeterminate bush	Sub-Saharan Africa	Ghana	Unknown	4,41	478	2
INCBN 00850	indeterminate climbing	Southern Europe	Greece	Landrace	4,13	479	4
INCBN 00704	determinate bush	Southern Europe	Italy	Landrace	4,08	480	2
INCBN 00177	indeterminate climbing	Southern Europe	Italy	Landrace	4,01	481	4
INCBN 03193	indeterminate climbing	Eastern Europe	Slovakia	Landrace	3,98	482	4
INCBN 03488	determinate bush	Unknown	unknown	Cultivar	3,94	483	2
INCBN 00264	determinate bush	America	Mexico	Unknown	3,87	484	2
INCBN 00118	determinate bush	Southern Europe	Italy	Landrace	3,86	485	2
INCBN 01669	determinate bush	Central America	Cuba	Landrace	3,85	486	2
INCBN 00195	indeterminate prostrate	Eastern Europe	Bulgaria	Landrace	3,80	487	4
INCBN 00138	indeterminate bush	Eastern Europe	Romania	Landrace	3,64	488	4
INCBN 09776	determinate climbing	Western Europe	Germany	Unknown	3,63	489	2
INCBN 00111	determinate climbing	Southern Europe	Spain	Landrace	3,60	490	4
INCBN 00265	indeterminate bush	South America	Colombia	Unknown	3,52	491	2
INCBN 00160	indeterminate climbing	Eastern Europe	Hungary	Landrace	3,41	492	2
INCBN 02757	indeterminate climbing	Southern Europe	Greece	Landrace	3,30	493	4
INCBN 01550	indeterminate prostrate	Central America	Cuba	Landrace	3,18	494	2
INCBN 02530	determinate bush	Western Europe	Germany	Cultivar	3,14	495	2
INCBN 01752	indeterminate climbing	Eastern Europe	Georgia	Landrace	3,13	496	2
INCBN 01632	indeterminate climbing	Central America	Cuba	Landrace	3,10	497	4
INCBN 08930	indeterminate prostrate	Sub-Saharan Africa	Zambia	Unknown	3,03	498	2
INCBN 01728	indeterminate prostrate	Eastern Europe	Georgia	Landrace	2,96	499	2
INCBN 00413	determinate climbing	Southern Europe	Spain	Landrace	2,91	500	4
INCBN 07105	determinate bush	Southern Europe	Spain	Unknown	2,87	501	2
INCBN 07173	indeterminate climbing	Southern Europe	Spain	Unknown	2,77	502	2
INCBN 06383	indeterminate climbing	Eastern Europe	Albania	Unknown	2,67	503	2
INCBN_10637	indeterminate climbing	Unknown	unknown	Unknown	2,15	504	2
INCBN 03533	indeterminate climbing	North America	USA	Cultivar	1,90	505	4
INCBN 01539	determinate bush	Central America	Cuba	Landrace	1,34	506	2
INCBN 01640	indeterminate bush	Central America	Cuba	Landrace	1,00	507	2

Table S5, Characteristics of the 19 R-core lines in HCPC Cluster 4 used in multi-environment analyses,

Line	PH	GEO REGION	Country	Biological status	HCPC Cluster
INCBN 00111	determinate climbing	Southern Europe	Spain	Landrace	4
INCBN 00143	indeterminate prostrate	Western Europe	Austria	Landrace	4
INCBN 00413	determinate climbing	Southern Europe	Spain	Landrace	4
INCBN 00433	indeterminate climbing	Southern Europe	Greece	Landrace	4
INCBN 00444	indeterminate climbing	Southern Europe	Italy	Landrace	4
INCBN 00474	indeterminate climbing	Eastern Europe	Albania	Landrace	4
INCBN 01323	indeterminate prostrate	Western Europe	Austria	Landrace	4
INCBN 01359	indeterminate climbing	Western Europe	Austria	Landrace	4
INCBN 02002	indeterminate climbing	Eastern Europe	Georgia	Landrace	4
INCBN 02066	indeterminate climbing	Eastern Europe	Georgia	Landrace	4
INCBN 02821	indeterminate climbing	Southern Europe	Italy	Landrace	4
INCBN 02842	indeterminate climbing	Southern Europe	Italy	Landrace	4
INCBN 02957	indeterminate climbing	Southern Europe	Italy	Landrace	4
INCBN 03046	indeterminate climbing	Southern Europe	Italy	Landrace	4
INCBN 03223	indeterminate climbing	Eastern Europe	Slovakia	Landrace	4
INCBN 03229	indeterminate prostrate	Eastern Europe	Slovakia	Landrace	4
INCBN 03273	indeterminate climbing	Eastern Europe	Slovakia	Landrace	4
INCBN 03286	indeterminate climbing	Eastern Europe	Slovakia	Landrace	4
INCBN 03300	indeterminate climbing	Eastern Europe	Slovakia	Landrace	4

Table S6 A. List of *Phaseolus vulgaris* lines included in the phenotypic analyses (507 lines).

2021				2022	
**Increase ID**	**DOI number of SSD line**	**Increase ID**	**DOI number of SSD line**	**Increase ID**	**DOI number of SSD line**
INCBN_00003	10.18730/H7P1D	INCBN_01377	10.18730/WK02B	INCBN_00041	10.18730/H7Q8F
INCBN_00011	10.18730/H7P9N	INCBN_01385	10.18730/WK0AK	INCBN_00108	10.18730/H7XV4
INCBN_00019	10.18730/H7PHX	INCBN_01409	10.18730/WK126	INCBN_00112	10.18730/H7Y09
INCBN_00020	10.18730/H7PJY	INCBN_01417	10.18730/WK1AE	INCBN_00118	10.18730/H7Y6F
INCBN_00048	10.18730/H7QFP	INCBN_01423	10.18730/WK1GM	INCBN_00130	10.18730/H7YJV
INCBN_00051	10.18730/H7QJS	INCBN_01426	10.18730/WK1KQ	INCBN_00139	10.18730/H7YVU
INCBN_00052	10.18730/H7QKT	INCBN_01432	10.18730/WK1SX	INCBN_00146	10.18730/H7Z26
INCBN_00054	10.18730/H7QNW	INCBN_01434	10.18730/WK1VZ	INCBN_00152	10.18730/H7Z8C
INCBN_00055	10.18730/H7QPX	INCBN_01441	10.18730/WK221	INCBN_00157	10.18730/H7ZDH
INCBN_00057	10.18730/H7QRZ	INCBN_01526	10.18730/WK4QC	INCBN_00160	10.18730/H7ZGM
INCBN_00066	10.18730/H7R13	INCBN_01530	10.18730/WK4VG	INCBN_00174	10.18730/H7ZY$
INCBN_00072	10.18730/H7R79	INCBN_01553	10.18730/WK5J2	INCBN_00183	10.18730/H8098
INCBN_00075	10.18730/H7RAC	INCBN_01609	10.18730/WK7AN	INCBN_00184	10.18730/H80A9
INCBN_00081	10.18730/H7RHK	INCBN_01620	10.18730/WK7N*	INCBN_00187	10.18730/H80FE
INCBN_00089	10.18730/H7RTW	INCBN_01632	10.18730/WK817	INCBN_00188	10.18730/H80GF
INCBN_00090	10.18730/H7RVX	INCBN_01740	10.18730/WKBD4	INCBN_00194	10.18730/H80PN
INCBN_00096	10.18730/H7S2U	INCBN_01764	10.18730/WKC5W	INCBN_00230	10.18730/H7SC9
INCBN_00111	10.18730/H7XY7	INCBN_01767	10.18730/WKC8Z	INCBN_00231	10.18730/H7SDA
INCBN_00113	10.18730/H7Y1A	INCBN_01780	10.18730/WKCN7	INCBN_00247	10.18730/H7SXT
INCBN_00114	10.18730/H7Y2B	INCBN_01806	10.18730/WKDF~	INCBN_00260	10.18730/H7TA2
INCBN_00116	10.18730/H7Y4D	INCBN_01807	10.18730/WKDG$	INCBN_00264	10.18730/H7TE6
INCBN_00120	10.18730/H7Y8H	INCBN_01808	10.18730/WKDH=	INCBN_00265	10.18730/H7TF7
INCBN_00121	10.18730/H7Y9J	INCBN_01881	10.18730/WKFT$	INCBN_00291	10.18730/H7V9~
INCBN_00123	10.18730/H7YBM	INCBN_01889	10.18730/WKG25	INCBN_00295	10.18730/H7VD0
INCBN_00124	10.18730/H7YCN	INCBN_01898	10.18730/WKGBE	INCBN_00302	10.18730/H7VM7
INCBN_00127	10.18730/H7YFR	INCBN_01914	10.18730/WKGVY	INCBN_00319	10.18730/H7W5R
INCBN_00128	10.18730/H7YGS	INCBN_01933	10.18730/WKHEC	INCBN_00327	10.18730/H7WD*
INCBN_00134	10.18730/H7YPZ	INCBN_01943	10.18730/WKHRP	INCBN_00331	10.18730/H7WHU
INCBN_00135	10.18730/H7YQ*	INCBN_01998	10.18730/WKKF3	INCBN_00341	10.18730/H7WV9
INCBN_00136	10.18730/H7YR~	INCBN_02002	10.18730/WKKK7	INCBN_00344	10.18730/H7WYC
INCBN_00137	10.18730/H7YS$	INCBN_02009	10.18730/WKKTE	INCBN_00346	10.18730/H7X0E
INCBN_00138	10.18730/H7YT=	INCBN_02011	10.18730/WKKWG	INCBN_00372	10.18730/H7XZ8
INCBN_00143	10.18730/H7YZ3	INCBN_02012	10.18730/WKKXH	INCBN_00375	10.18730/H80BA
INCBN_00145	10.18730/H7Z15	INCBN_02017	10.18730/WKM2P	INCBN_00384	10.18730/H81A4
INCBN_00147	10.18730/H7Z37	INCBN_02024	10.18730/WKM9X	INCBN_00389	10.18730/H81F9
INCBN_00148	10.18730/H7Z48	INCBN_02027	10.18730/WKMC*	INCBN_00394	10.18730/H81ME
INCBN_00149	10.18730/H7Z59	INCBN_02031	10.18730/WKMGU	INCBN_00398	10.18730/H81RJ
INCBN_00158	10.18730/H7ZEJ	INCBN_02036	10.18730/WKMN4	INCBN_00399	10.18730/H81SK
INCBN_00159	10.18730/H7ZFK	INCBN_02038	10.18730/WKMQ6	INCBN_00404	10.18730/H81YR
INCBN_00172	10.18730/H7ZW*	INCBN_02042	10.18730/WKMVA	INCBN_00425	10.18730/H82K8
INCBN_00173	10.18730/H7ZX~	INCBN_02052	10.18730/WKN5M	INCBN_00450	10.18730/H83C~
INCBN_00177	10.18730/H8032	INCBN_02054	10.18730/WKN7P	INCBN_00482	10.18730/H84CW
INCBN_00180	10.18730/H8065	INCBN_02058	10.18730/WKNBT	INCBN_00506	10.18730/H854F
INCBN_00195	10.18730/H80QP	INCBN_02059	10.18730/WKNCV	INCBN_00651	10.18730/WJ9CS
INCBN_00198	10.18730/H80TS	INCBN_02066	10.18730/WKNK$	INCBN_00698	10.18730/WJAV=
INCBN_00201	10.18730/H80XW	INCBN_02068	10.18730/WKNNU	INCBN_00704	10.18730/WJB14
INCBN_00202	10.18730/H80YX	INCBN_02070	10.18730/WKNQ1	INCBN_00824_A	10.18730/WJESD
INCBN_00203	10.18730/H80ZY	INCBN_02088	10.18730/WKP9K	INCBN_00824_B	10.18730/WJESD
INCBN_00206	10.18730/H814=	INCBN_02091	10.18730/WKPCP	INCBN_00837	10.18730/WJF6T
INCBN_00225	10.18730/H7S74	INCBN_02098	10.18730/WKPKX	INCBN_00842	10.18730/WJFBZ
INCBN_00256	10.18730/H7T6=	INCBN_02110	10.18730/WKPZ4	INCBN_00851	10.18730/WJFM3
INCBN_00284	10.18730/H7V2T	INCBN_02731	10.18730/WMAC~	INCBN_00858	10.18730/WJFVA
INCBN_00293	10.18730/H7VB=	INCBN_02734	10.18730/WMAFU	INCBN_00873	10.18730/WJGAS
INCBN_00297	10.18730/H7VF2	INCBN_02736	10.18730/WMAH1	INCBN_00950	10.18730/WJJQW
INCBN_00323	10.18730/H7W9W	INCBN_02737	10.18730/WMAJ2	INCBN_00969	10.18730/WJKAA
INCBN_00354	10.18730/H7X8P	INCBN_02746	10.18730/WMAVB	INCBN_01496	10.18730/WK3SK
INCBN_00378	10.18730/H813$	INCBN_02749	10.18730/WMAYE	INCBN_01524	10.18730/WK4NA
INCBN_00379	10.18730/H815U	INCBN_02757	10.18730/WMB6P	INCBN_01539	10.18730/WK54S
INCBN_00380	10.18730/H8160	INCBN_02759	10.18730/WMB8R	INCBN_01550	10.18730/WK5FU
INCBN_00381	10.18730/H8171	INCBN_02762	10.18730/WMBBV	INCBN_01579	10.18730/WK6CW
INCBN_00393	10.18730/H81KD	INCBN_02764	10.18730/WMBDX	INCBN_01629	10.18730/WK7Y4
INCBN_00395	10.18730/H81NF	INCBN_02765	10.18730/WMBEY	INCBN_01640	10.18730/WK89F
INCBN_00400	10.18730/H81TM	INCBN_02799	10.18730/WMCGV	INCBN_01654	10.18730/WK8QX
INCBN_00413	10.18730/H827~	INCBN_02821	10.18730/WMD6C	INCBN_01658	10.18730/WK8V~
INCBN_00429	10.18730/H82QC	INCBN_02822	10.18730/WMD7D	INCBN_01669	10.18730/WK967
INCBN_00430	10.18730/H82RD	INCBN_02828	10.18730/WMDDK	INCBN_01728	10.18730/WKB1X
INCBN_00431	10.18730/H82SE	INCBN_02830	10.18730/WMDFN	INCBN_01752	10.18730/WKBSG
INCBN_00432	10.18730/H82TF	INCBN_02833	10.18730/WMDJR	INCBN_01779	10.18730/WKCM6
INCBN_00433	10.18730/H82VG	INCBN_02836	10.18730/WMDNV	INCBN_01783	10.18730/WKCRA
INCBN_00435	10.18730/H82XJ	INCBN_02838	10.18730/WMDQX	INCBN_01804	10.18730/WKDDZ

INCBN_00436	10.18730/H82YK	INCBN_02842	10.18730/WMDV~	INCBN_01868	10.18730/WKFDN
INCBN_00437	10.18730/H82ZM	INCBN_02851	10.18730/WME45	INCBN_01992_A	10.18730/WKK9$
INCBN_00438	10.18730/H830N	INCBN_02853	10.18730/WME67	INCBN_01992_B	10.18730/WKK9$
INCBN_00439	10.18730/H831P	INCBN_02855	10.18730/WME89	INCBN_02226	10.18730/WKTK9
INCBN_00440	10.18730/H832Q	INCBN_02856	10.18730/WME9A	INCBN_02240	10.18730/WKV1Q
INCBN_00441	10.18730/H833R	INCBN_02857	10.18730/WMEAB	INCBN_02410	10.18730/WM0B8
INCBN_00443	10.18730/H835T	INCBN_02859	10.18730/WMECD	INCBN_02530	10.18730/WM43H
INCBN_00444	10.18730/H836V	INCBN_02860	10.18730/WMEDE	INCBN_02760	10.18730/WMB9S
INCBN_00445	10.18730/H837W	INCBN_02863	10.18730/WMEGH	INCBN_02761	10.18730/WMBAT
INCBN_00446	10.18730/H838X	INCBN_02864	10.18730/WMEHJ	INCBN_02771_A	10.18730/WMBMU
INCBN_00448	10.18730/H83AZ	INCBN_02888	10.18730/WMF95	INCBN_02771_B	10.18730/WMBMU
INCBN_00449	10.18730/H83B*	INCBN_02891	10.18730/WMFC8	INCBN_02801	10.18730/WMCJX
INCBN_00451	10.18730/H83D$	INCBN_02894	10.18730/WMFFB	INCBN_02823	10.18730/WMD8E
INCBN_00457	10.18730/H83K3	INCBN_02895	10.18730/WMFGC	INCBN_02832	10.18730/WMDHQ
INCBN_00459	10.18730/H83N5	INCBN_02897	10.18730/WMFJE	INCBN_02841	10.18730/WMDT*
INCBN_00460	10.18730/H83P6	INCBN_02917	10.18730/WMG6$	INCBN_02849	10.18730/WME23
INCBN_00461	10.18730/H83Q7	INCBN_02932	10.18730/WMGNC	INCBN_02872	10.18730/WMEST
INCBN_00462	10.18730/H83R8	INCBN_02933	10.18730/WMGPD	INCBN_02882	10.18730/WMF3U
INCBN_00463	10.18730/H83S9	INCBN_02948	10.18730/WMH5W	INCBN_03023	10.18730/WMKGX
INCBN_00464	10.18730/H83TA	INCBN_02950	10.18730/WMH7Y	INCBN_03055	10.18730/WMMGR
INCBN_00465	10.18730/H83VB	INCBN_02952	10.18730/WMH9*	INCBN_03101	10.18730/WMNY~
INCBN_00469	10.18730/H83ZF	INCBN_02953	10.18730/WMHA~	INCBN_03163	10.18730/WMQWN
INCBN_00470	10.18730/H840G	INCBN_02954	10.18730/WMHB$	INCBN_03188	10.18730/WMRN9
INCBN_00471	10.18730/H841H	INCBN_02957	10.18730/WMHE0	INCBN_03190_A	10.18730/WMRQB
INCBN_00472	10.18730/H842J	INCBN_02960	10.18730/WMHH3	INCBN_03190_B	10.18730/WMRQB
INCBN_00473	10.18730/H843K	INCBN_02962	10.18730/WMHK5	INCBN_03196	10.18730/WMRXH
INCBN_00474	10.18730/H844M	INCBN_02972	10.18730/WMHXF	INCBN_03220	10.18730/WMSN4
INCBN_00477	10.18730/H847Q	INCBN_02980	10.18730/WMJ5Q	INCBN_03228	10.18730/WMSXC
INCBN_00478	10.18730/H848R	INCBN_02994	10.18730/WMJK0	INCBN_03366	10.18730/WMY72
INCBN_00479	10.18730/H849S	INCBN_03015	10.18730/WMK8N	INCBN_03383	10.18730/WMYRK
INCBN_00480	10.18730/H84AT	INCBN_03028	10.18730/WMKN$	INCBN_03426	10.18730/WN03S
INCBN_00483	10.18730/H84DX	INCBN_03034	10.18730/WMKV3	INCBN_03488	10.18730/WN21D
INCBN_00484	10.18730/H84EY	INCBN_03046	10.18730/WMM7F	INCBN_03509	10.18730/WN2P$
INCBN_00485	10.18730/H84FZ	INCBN_03047	10.18730/WMM8G	INCBN_06383	10.18730/WPCD0
INCBN_00486	10.18730/H84G*	INCBN_03048	10.18730/WMM9H	INCBN_06385	10.18730/WPCF2
INCBN_00488	10.18730/H84J$	INCBN_03053	10.18730/WMMEP	INCBN_06440	10.18730/WPE6M
INCBN_00489	10.18730/H84K=	INCBN_03059	10.18730/WMMMW	INCBN_06451	10.18730/WPEHZ
INCBN_00491	10.18730/H84N0	INCBN_03061	10.18730/WMMPY	INCBN_06452	10.18730/WPEJ*
INCBN_00494	10.18730/H84R3	INCBN_03085	10.18730/WMNEH	INCBN_06455	10.18730/WPEN=
INCBN_00495	10.18730/H84S4	INCBN_03193	10.18730/WMRTE	INCBN_06458	10.18730/WPER1
INCBN_00496	10.18730/H84T5	INCBN_03194	10.18730/WMRVF	INCBN_06473	10.18730/WPF7G
INCBN_00497	10.18730/H84V6	INCBN_03198	10.18730/WMRZK	INCBN_06476	10.18730/WPFAK
INCBN_00502	10.18730/H850B	INCBN_03201	10.18730/WMS2P	INCBN_06503	10.18730/WPG59
INCBN_00504	10.18730/H852D	INCBN_03207	10.18730/WMS8W	INCBN_06540	10.18730/WPHA9
INCBN_00505	10.18730/H853E	INCBN_03210	10.18730/WMSBZ	INCBN_06547	10.18730/WPHHG
INCBN_00507	10.18730/H855G	INCBN_03213	10.18730/WMSE$	INCBN_06563	10.18730/WPJ1*
INCBN_00508	10.18730/H856H	INCBN_03222	10.18730/WMSQ6	INCBN_06565	10.18730/WPJ3$
INCBN_00509	10.18730/H857J	INCBN_03223	10.18730/WMSR7	INCBN_06604	10.18730/WPKAU
INCBN_00510	10.18730/H858K	INCBN_03229	10.18730/WMSYD	INCBN_06631	10.18730/WPM5T
INCBN_00512	10.18730/H85AN	INCBN_03237	10.18730/WMT6N	INCBN_06659	10.18730/WPN1H
INCBN_00513	10.18730/H85BP	INCBN_03241	10.18730/WMTAS	INCBN_06666	10.18730/WPN8R
INCBN_00514	10.18730/H85CQ	INCBN_03248	10.18730/WMTH*	INCBN_06690	10.18730/WPP0B
INCBN_00515	10.18730/H85DR	INCBN_03257	10.18730/WMTT4	INCBN_06704	10.18730/WPPES
INCBN_00516	10.18730/H85ES	INCBN_03260	10.18730/WMTX7	INCBN_06708	10.18730/WPPJX
INCBN_00519	10.18730/H85HW	INCBN_03261	10.18730/WMTY8	INCBN_06736	10.18730/WPQEM
INCBN_00520	10.18730/H85JX	INCBN_03262	10.18730/WMTZ9	INCBN_06779	10.18730/WPRST
INCBN_00817	10.18730/WJEJ6	INCBN_03263	10.18730/WMV0A	INCBN_06809	10.18730/WPSQK
INCBN_00826	10.18730/WJEVF	INCBN_03264	10.18730/WMV1B	INCBN_06858	10.18730/WPV8Z
INCBN_00833	10.18730/WJF2P	INCBN_03267	10.18730/WMV4E	INCBN_06881	10.18730/WPVZH
INCBN_00840	10.18730/WJF9X	INCBN_03268	10.18730/WMV5F	INCBN_06884	10.18730/WPW2M
INCBN_00848	10.18730/WJFH0	INCBN_03273	10.18730/WMVAM	INCBN_06892	10.18730/WPWAW
INCBN_00850	10.18730/WJFK2	INCBN_03275	10.18730/WMVCP	INCBN_07070	10.18730/WQ1WN
INCBN_00874	10.18730/WJGBT	INCBN_03277	10.18730/WMVER	INCBN_07105	10.18730/WQ2ZK
INCBN_00875	10.18730/WJGCV	INCBN_03281	10.18730/WMVJW	INCBN_07147	10.18730/WQ49R
INCBN_00895	10.18730/WJH0A	INCBN_03286	10.18730/WMVQ~	INCBN_07161	10.18730/WQ4Q1
INCBN_00915	10.18730/WJHMY	INCBN_03288	10.18730/WMVS=	INCBN_07173	10.18730/WQ53D
INCBN_00920	10.18730/WJHS=	INCBN_03289	10.18730/WMVTU	INCBN_07222	10.18730/WQ6MS
INCBN_00924	10.18730/WJHX2	INCBN_03291	10.18730/WMVW1	INCBN_07388	10.18730/WQBT6
INCBN_00974	10.18730/WJKFF	INCBN_03292	10.18730/WMVX2	INCBN_07540	10.18730/WQGJA
INCBN_00975	10.18730/WJKGG	INCBN_03294	10.18730/WMVZ4	INCBN_08834	10.18730/WRS09
INCBN_01194	10.18730/WJTBD	INCBN_03298	10.18730/WMW38	INCBN_08912	10.18730/WRVED
INCBN_01199	10.18730/WJTGJ	INCBN_03299	10.18730/WMW49	INCBN_08923	10.18730/WRVSR
INCBN_01202	10.18730/WJTKN	INCBN_03300	10.18730/WMW5A	INCBN_08930	10.18730/WRW0Z
INCBN_01203	10.18730/WJTMP	INCBN_03301	10.18730/WMW6B	INCBN_09154	10.18730/WS30~

INCBN_01212	10.18730/WJTXZ	INCBN_03305	10.18730/WMWAF	INCBN_09226	10.18730/WS58Z
INCBN_01215	10.18730/WJV0$	INCBN_03309	10.18730/WMWEK	INCBN_09250	10.18730/WS60J
INCBN_01222	10.18730/WJV74	INCBN_03385	10.18730/WMYTN	INCBN_09474	10.18730/WSD0M
INCBN_01237	10.18730/WJVPK	INCBN_03435	10.18730/WN0C$	INCBN_09634	10.18730/WSHYY
INCBN_01240	10.18730/WJVSP	INCBN_03445	10.18730/WN0P7	INCBN_09635	10.18730/WSHZZ
INCBN_01241	10.18730/WJVTQ	INCBN_03479	10.18730/WN1R4	INCBN_09693	10.18730/WSKSF
INCBN_01258	10.18730/WJWB3	INCBN_03512	10.18730/WN2S0	INCBN_09695	10.18730/WSKVH
INCBN_01268	10.18730/WJWND	INCBN_03533	10.18730/WN3EN	INCBN_09758	10.18730/WSNR4
INCBN_01284	10.18730/WJX5X	INCBN_03536	10.18730/WN3HR	INCBN_09776	10.18730/WSPAP
INCBN_01285	10.18730/WJX6Y	INCBN_09985	10.18730/SBC1E	INCBN_09929	10.18730/WSV3V
INCBN_01286	10.18730/WJX7Z	INCBN_10026	10.18730/SC1YC	INCBN_09935	10.18730/WSV9~
INCBN_01288	10.18730/WJX9~			INCBN_09971	10.18730/WSWD*
INCBN_01289	10.18730/WJXA$			INCBN_10028	10.18730/SC20E
INCBN_01297	10.18730/WJXJ5			INCBN_10361	10.18730/1021W*
INCBN_01298	10.18730/WJXK6			INCBN_10368	10.18730/1022A9
INCBN_01299	10.18730/WJXM7			INCBN_10370	10.18730/1022HG
INCBN_01300	10.18730/WJXN8			INCBN_10371	10.18730/1022JH
INCBN_01305	10.18730/WJXTD			INCBN_10375	10.18730/10230Z
INCBN_01306	10.18730/WJXVE			INCBN_10378	10.18730/102221
INCBN_01309	10.18730/WJXYH			INCBN_10380	10.18730/1022ED
INCBN_01310	10.18730/WJXZJ			INCBN_10381	10.18730/10235U
INCBN_01313	10.18730/WJY2N			INCBN_10382	10.18730/1VKPN4
INCBN_01323	10.18730/WJYCZ			INCBN_10383	10.18730/1VKPP5
INCBN_01328	10.18730/WJYHU			INCBN_10384	10.18730/1VKPQ6
INCBN_01333	10.18730/WJYP4			INCBN_10385	10.18730/1VKPR7
INCBN_01335	10.18730/WJYR6			INCBN_10386	10.18730/1VKPS8
INCBN_01337	10.18730/WJYT8			INCBN_10634	10.18730/1VKPT9
INCBN_01359	10.18730/WJZGY			INCBN_10635	10.18730/1VKPVA
INCBN_01369	10.18730/WJZT3			INCBN_10637	10.18730/1VKPWB
INCBN_01373	10.18730/WJZY7			INCBN_10641	10.18730/1VKPXC
INCBN_01374	10.18730/WJZZ8			INCBN_10642	10.18730/1VKPYD
INCBN_01376	10.18730/WK01A			INCBN_10645	10.18730/1VKQ0F

This table lists the 507 INCREASE R-core records of *Phaseolus vulgaris* that reached flowering, produced complete phenotypic data, and were included in the analyses. Four lines evaluated in 2022 were grown twice and are indicated with the suffixes _A and _B; after excluding these duplicate records, 503 unique lines remain. In total, 447 lines were evaluated in 2021 and 194 were grown in 2022.

Table S6 B. *Phaseolus vulgaris* lines excluded from phenotypic analyses.

2021				2022	
**Increase ID**	**DOI number of SSD line**	**Increase ID**	**DOI number of SSD line**	**Increase ID**	**DOI number of SSD line**
INCBN_00002	10.18730/H7P0C	INCBN_00171	10.18730/H7ZVZ	INCBN_00181	10.18730/H8076
INCBN_00006	10.18730/H7P4G	INCBN_00174	10.18730/H7ZY$	INCBN_00248	10.18730/H7SYV
INCBN_00007	10.18730/H7P5H	INCBN_00175	10.18730/H7ZZ=	INCBN_00429	10.18730/H82QC
INCBN_00016	10.18730/H7PET	INCBN_00184	10.18730/H80A9	INCBN_00452	10.18730/H83E=
INCBN_00017	10.18730/H7PFV	INCBN_00186	10.18730/H80ED	INCBN_00948	10.18730/WJJNT
INCBN_00018	10.18730/H7PGW	INCBN_00189	10.18730/H80HG	INCBN_00972	10.18730/WJKDD
INCBN_00021	10.18730/H7PKZ	INCBN_00196	10.18730/H80RQ	INCBN_01181	10.18730/WJSY0
INCBN_00026	10.18730/H7PRU	INCBN_00200	10.18730/H80WV	INCBN_01221	10.18730/WJV63
INCBN_00027	10.18730/H7PS0	INCBN_00205	10.18730/H811*	INCBN_01226	10.18730/WJVB8
INCBN_00037	10.18730/H7Q3A	INCBN_00226	10.18730/H7S85	INCBN_01314	10.18730/WJY3P
INCBN_00038	10.18730/H7Q4B	INCBN_00227	10.18730/H7S96	INCBN_01347	10.18730/WJZ4J
INCBN_00039	10.18730/H7Q6D	INCBN_00236	10.18730/H7SJF	INCBN_01703	10.18730/WKA84
INCBN_00044	10.18730/H7QBJ	INCBN_00242	10.18730/H7SRN	INCBN_01724	10.18730/WKAXS
INCBN_00047	10.18730/H7QEN	INCBN_00246	10.18730/H7SWS	INCBN_01987	10.18730/WKK4X
INCBN_00056	10.18730/H7QQY	INCBN_00250	10.18730/H7T0X	INCBN_01993	10.18730/WKKA=
INCBN_00059	10.18730/H7QT~	INCBN_00258	10.18730/H7T80	INCBN_02023	10.18730/WKM8W
INCBN_00060	10.18730/H7QV$	INCBN_00259	10.18730/H7T91	INCBN_02742	10.18730/WMAQ7
INCBN_00062	10.18730/H7QXU	INCBN_00271	10.18730/H7TND	INCBN_02806	10.18730/WMCQ$
INCBN_00063	10.18730/H7QY0	INCBN_00272	10.18730/H7TPE	INCBN_02811	10.18730/WMCW2
INCBN_00064	10.18730/H7QZ1	INCBN_00274	10.18730/H7TRG	INCBN_02981	10.18730/WMJ6R
INCBN_00065	10.18730/H7R02	INCBN_00276	10.18730/H7TTJ	INCBN_02991	10.18730/WMJG$
INCBN_00068	10.18730/H7R35	INCBN_00289	10.18730/H7V7Z	INCBN_03105	10.18730/WMP20
INCBN_00070	10.18730/H7R57	INCBN_00294	10.18730/H7VCU	INCBN_03146	10.18730/WMQB4
INCBN_00076	10.18730/H7RBD	INCBN_00296	10.18730/H7VE1	INCBN_03150	10.18730/WMQF8
INCBN_00077	10.18730/H7RCE	INCBN_00299	10.18730/H7VH4	INCBN_03302	10.18730/WMW7C
INCBN_00078	10.18730/H7REG	INCBN_00303	10.18730/H7VN8	INCBN_03368	10.18730/WMY94
INCBN_00079	10.18730/H7RFH	INCBN_00329	10.18730/H7WF$	INCBN_07163	10.18730/WQ4S3
INCBN_00080	10.18730/H7RGJ	INCBN_00339	10.18730/H7WS7	INCBN_10644	10.18730/1VKPZE
INCBN_00082	10.18730/H7RJM	INCBN_00340	10.18730/H7WT8		
INCBN_00083	10.18730/H7RKN	INCBN_00349	10.18730/H7X3H		
INCBN_00084	10.18730/H7RMP	INCBN_00447	10.18730/H839Y		
INCBN_00085	10.18730/H7RNQ	INCBN_00466	10.18730/H83WC		
INCBN_00087	10.18730/H7RQS	INCBN_00467	10.18730/H83XD		
INCBN_00088	10.18730/H7RRT	INCBN_00468	10.18730/H83YE		
INCBN_00091	10.18730/H7RWY	INCBN_00490	10.18730/H84MU		
INCBN_00094	10.18730/H7S0$	INCBN_00493	10.18730/H84Q2		
INCBN_00107	10.18730/H7XT3	INCBN_00498	10.18730/H84W7		
INCBN_00133	10.18730/H7YNY	INCBN_00499	10.18730/H84X8		
INCBN_00141	10.18730/H7YX1	INCBN_00500	10.18730/H84Y9		
INCBN_00144	10.18730/H7Z04	INCBN_00501	10.18730/H84ZA		
INCBN_00146	10.18730/H7Z26	INCBN_00503	10.18730/H851C		
INCBN_00150	10.18730/H7Z6A	INCBN_00511	10.18730/H859M		
INCBN_00151	10.18730/H7Z7B	INCBN_00518	10.18730/H85GV		
INCBN_00152	10.18730/H7Z8C	INCBN_00521	10.18730/H85KY		
INCBN_00153	10.18730/H7Z9D	INCBN_00815	10.18730/WJEG4		
INCBN_00154	10.18730/H7ZAE	INCBN_00816	10.18730/WJEH5		
INCBN_00155	10.18730/H7ZBF	INCBN_00824	10.18730/WJESD		
INCBN_00156	10.18730/H7ZCG	INCBN_00841	10.18730/WJFAY		
INCBN_00157	10.18730/H7ZDH	INCBN_01278	10.18730/WJWZQ		
INCBN_00160	10.18730/H7ZGM	INCBN_01320	10.18730/WJY9W		
INCBN_00161	10.18730/H7ZHN	INCBN_01322	10.18730/WJYBY		
INCBN_00162	10.18730/H7ZJP	INCBN_01330	10.18730/WJYK1		
INCBN_00163	10.18730/H7ZKQ	INCBN_01942	10.18730/WKHQN		
INCBN_00164	10.18730/H7ZMR	INCBN_02014	10.18730/WKKZK		
INCBN_00165	10.18730/H7ZNS	INCBN_02756	10.18730/WMB5N		
INCBN_00166	10.18730/H7ZPT	INCBN_02763	10.18730/WMBCW		
INCBN_00167	10.18730/H7ZQV	INCBN_02771	10.18730/WMBMU		
INCBN_00168	10.18730/H7ZRW	INCBN_02804	10.18730/WMCN*		
INCBN_00169	10.18730/H7ZSX	INCBN_10148	10.18730/SC5NM		
INCBN_00170	10.18730/H7ZTY				

This table lists the 138 INCREASE R-core lines excluded from phenotypic analyses because they did not reach reproductive development (either did not germinate or failed to flower). Initially, a total of 147 lines failed to reach flowering; however, nine of these were successfully phenotyped after repeated plantings and were included in the final dataset. Exclusions were based solely on developmental failure and were not related to phenotypic performance.

Table S6 C. Subset of *Phaseolus vulgaris* lines evaluated across two growing seasons.

Evaluated lines		Excluded lines	
**Increase ID**	**DOI number of SSD lines**	**Increase ID**	**DOI number of SSD lines**
INCBN_00111	10.18730/H7XY7	INCBN_00146	10.18730/H7Z26
INCBN_00143	10.18730/H7YZ3	INCBN_00152	10.18730/H7Z8C
INCBN_00413	10.18730/H827~	INCBN_00157	10.18730/H7ZDH
INCBN_00433	10.18730/H82VG	INCBN_00160	10.18730/H7ZGM
INCBN_00444	10.18730/H836V	INCBN_00174	10.18730/H7ZY$
INCBN_00474	10.18730/H844M	INCBN_00184	10.18730/H80A9
INCBN_01323	10.18730/WJYCZ	INCBN_00429	10.18730/H82QC
INCBN_01359	10.18730/WJZGY		
INCBN_02002	10.18730/WKKK7		
INCBN_02066	10.18730/WKNK$		
INCBN_02821	10.18730/WMD6C		
INCBN_02842	10.18730/WMDV~		
INCBN_02957	10.18730/WMHE0		
INCBN_03046	10.18730/WMM7F		
INCBN_03223	10.18730/WMSR7		
INCBN_03229	10.18730/WMSYD		
INCBN_03273	10.18730/WMVAM		
INCBN_03286	10.18730/WMVQ~		
INCBN_03300	10.18730/WMW5A		

This table lists the 26 *Phaseolus vulgaris* lines evaluated during the 2021 and 2022 growing seasons. Seven lines reached flowering in only one year and were therefore excluded from multi-environment analyses. The remaining 19 lines produced complete yield data in both seasons and were used for multi-environment analyses of yield performance, yield stability, and genotype × environment interaction.

Tables S7 A. Quantitative trait descriptors used in the INCREASE common bean R-core phenotyping.

Parameter group	Descriptors	ID code	Unite
Emergence	Days to emergence	DE	Days
	Emerged plants	EP	Number
Flowering	Days to beginning of flowering (from sowing)	DBF	Days
	Days to maximum flowering (from sowing)	DMF	Days
	Days to the end of flowering	DEF	Days
Pod development and physiological maturity	Days to pod formation (from sowing)	DPF	Days
	Full maturity	FM	Days
	Days to harvest (from sowing)	DH	Days
	Number of plants with pods per plot	NPP	Number
	Pod length	PL	cm
Pod and seed production	Pod width	PW	cm
	Weight of ten dry pods per plot	WT	g
	Number of seeds in ten dry pods per plot	NST	Number
	Weight of total seeds in ten dry pods per plot	WTS	g
	1000-seed mass	W1000S	g
	Total number of seeds	TNS	Number
	Total seed mass	TSM	g
	Useless seed mass	US	g
	Plant canopy length	PCL	cm
	Stem diameter	SD	mm

Tables S7 B. Qualitative trait descriptors used in the INCREASE common bean R-core phenotyping.

Descriptors	ID code	Descriptor state	Descriptors	ID code	Descriptor state
Hypocotyl pigmentation	HP	Purple, Green, Other	Abiotic stress: high temperature	ASHT	Absent, Present
Leaf color: chlorophyll	LCC	Pale green, Medium green, Dark green	Other abiotic stress	OAS	Absent, Present
Leaf color: anthocyanin	LCA	Absent, Present	Pests: thrips	PTR	Absent, Present
Leaf persistence	LP	All leaves dropped, Intermediate, All leaves persistent	Pests: aphid	PAPH	Absent, Present
Leaf shape	LSH	LSH Triangular, Quadrangular, Round	Pests: flea beetle	PFLE	Absent, Present
Flower: color of standard	FCS	White, Greenish, Pink, Light purple, Purple, Dark purple, White with purple spots, White with red veins, White with green spots, Pink with green spots, Light purple with green spots, Red, Greenish with purple spots, Other	Pests: *Scaphoideus titanus*	PSCA	Absent, Present
Flower: color of wings	FCW	White, Greenish, Pink, Light purple, Purple, Dark purple, White/light purple, White with red veins, Other	Other pests	OPE	Absent, Present
Pod cross-section	PCS	Very flat, Pear shape, Round elliptic, Figure of eight, Other	Fungi present	FP	Absent, Present
Pod curvature	PC	Straight, Slightly curved, Curved, Recurving	Fungi: anthracnose	FANS	Absent, Present
Pod color at physiological maturity	PCPM	Light yellow, Gold yellow/dark yellow, Light green/grey green, Green/dark green, Red, Purple, Other	Fungi: root rot	FRR	Absent, Present
Pattern of pod pigmentation at physiological maturity	PPPM	None, Speckled, Mottled, Striped, Covered/coated, Other	Fungi: *Ascochyta*	FAS	Absent, Present
Pod wall fiber	PWF	Strongly contracting, Leathery podded, Excessive shattering	Fungi: rust	FRUS	Absent, Present
Pod color on fully expanded immature pods	PCFE	Light yellow, Golden yellow/dark yellow, Green/dark green, Light green/grey green, Red, Purple, Other	Fungi: angular leaf spot	FANO	Absent, Present
Pod suture string at technological maturity	PSS	Stringless, Few strings, Moderately stringy, Very stringy	Fungi: *Alternaria*	FALT	Absent, Present
Plant determinacy	PD	Determinate, Indeterminate	Fungi: other	FOT	Absent, Present
Plant growth habit	PH	Determinate bush, Indeterminate bush, Indeterminate prostrate, Indeterminate climbing, Determinate climbing	Bacteria and virus symptoms	BV	Absent, Present
Disease present	DPR	Absent, Present, Unsure	Virus: bean common mosaic necrosis virus (BCMNV)	BMN	Absent, Present
Stress susceptibility	SSU	Absent, Present, Unsure			
Abiotic stress: low temperature	ASLT	Absent, Present	Virus: bean common mosaic virus (BCMV)	BMV	Absent, Present

Table S8 A. Quantitative traits included in the selection index (SI), their assigned weights, and breeding-based justification

Trait code	Trait	Weight	Direction in SI	Brief justification
DE	Days to emergence	-0.05	Lower preferred	Earlier emergence was preferred.
EP	Emerged plants	0.10	Higher preferred	Higher emergence indicates better establishment and stand uniformity.
DBF	Days to beginning of flowering	-0.10	Lower preferred	Earlier flowering was preferred.
DMF	Days to maximum flowering	-0.10	Lower preferred	Earlier peak flowering was preferred.
DEF	Days to end of flowering	-0.05	Lower preferred	Long flowering duration was slightly penalized.
DPF	Days to pod formation	-0.05	Lower preferred	Earlier pod formation was preferred.
PCL	Plant canopy length	0.00	Neutral	Included descriptively but not weighted in the SI.
SD	Stem diameter	0.20	Higher preferred	Used as an indicator of plant vigor and structural strength.
FM	Full maturity	0.70	Higher preferred	Successful maturity was considered important for final productivity.
DH	Days to harvest	-0.05	Lower preferred	Longer crop duration was slightly penalized.
NPP	Number of plants with pods	0.90	Higher preferred	Important yield-related trait reflecting reproductive success.
PL	Pod length	0.10	Higher preferred	Minor positive contribution as a pod morphology trait.
PW	Pod width	0.10	Higher preferred	Minor positive contribution as a pod development trait.
TSM	Total seed mass	0.90	Higher preferred	One of the main traits contributing to yield performance.
US	Unfilled seeds / useless seed mass	-0.20	Lower preferred	Penalized because poor seed filling reduces effective yield.
BAS	Bruchid-attacked seeds	-0.20	Lower preferred	Penalized because seed damage reduces quality and usable yield.
TNS	Total number of seeds	0.70	Higher preferred	Important yield component.
W1000S	1000-seed weight	0.40	Higher preferred	Included as an indicator of seed size and agronomic value.
WT	Weight of ten dry pods per plots	0.05	Higher preferred	Minor contribution as a pod productivity trait.
NST	Number of seeds in ten dry pods per plot	0.20	Higher preferred	Moderately weighted as a seed set component.
WTS	Weight of total seeds in ten dry pods per plot	0.40	Higher preferred	Moderately weighted as a localized seed yield trait.

Table S8 B. Qualitative traits included in the selection index (SI), their assigned weights, and breeding-based justification

Trait code	Trait	Weight	Direction in SI	Brief justification
HP	Hypocotyl pigmentation	0.00	Neutral	Recorded descriptively but not weighted in the SI.
LCC	Leaf colour: chlorophyll	0.05	Presence/state favored	Slight positive contribution as a general indicator of healthy plant appearance.
LCA	Leaf colour: anthocyanin	0.05	Presence/state favored	Slight positive contribution within the phenotypic scoring scheme used.
FCS	Flower colour of standard	0.00	Neutral	Recorded descriptively but not weighted in the SI.
FCW	Flower colour of wings	0.00	Neutral	Recorded descriptively but not weighted in the SI.
PCS	Pod cross-section	0.30	Preferred state favored	Included as a positive pod morphology trait.
PC	Pod curvature	0.40	Preferred state favored	Given a relatively stronger contribution among pod morphology traits.
PCPM	Pod colour at physiological maturity	0.05	Preferred state favored	Minor contribution within pod phenotype assessment.
PPPM	Pattern of pod pigmentation at physiological maturity	0.05	Preferred state favored	Minor contribution within pod phenotype assessment.
PSS	Pod suture string	0.00	Neutral	Recorded descriptively but not weighted in the SI.
PWF	Pod wall fibre	0.00	Neutral	Recorded descriptively but not weighted in the SI.
LP	Leaf persistence	0.05	Preferred state favored	Slight positive contribution.
LSH	Leaf shape	0.00	Neutral	Recorded descriptively but not weighted in the SI.
PD	Plant determinacy	0.00	Neutral	Included descriptively but not directly weighted.
PH	Plant habit	0.00	Neutral	Included descriptively but not directly weighted.
DPR	Diseases present	-0.90	Absence preferred	Strong penalty because disease presence reduces agronomic value.
SSU	Stress susceptibility	-0.90	Absence preferred	Strong penalty because stress sensitivity is undesirable in breeding material.
ASLT	Abiotic stress: low temperature	-0.10	Absence preferred	Mild penalty for low-temperature stress symptoms.
ASHT	Abiotic stress: high temperature	-0.10	Absence preferred	Mild penalty for high-temperature stress symptoms.
OAS	Other abiotic stress	-0.10	Absence preferred	Mild penalty for additional abiotic stress symptoms.
PTR	Thrips	-0.10	Absence preferred	Mild penalty for pest incidence.
PAPH	Aphid	-0.10	Absence preferred	Mild penalty for pest incidence.
PFLE	Flea beetle	-0.10	Absence preferred	Mild penalty for pest incidence.
PSCA	*Scaphoideus titanus*	-0.10	Absence preferred	Mild penalty for pest incidence.
OPE	Other pests	-0.10	Absence preferred	Mild penalty for other pest damage.
FP	Fungi present	-0.10	Absence preferred	Mild penalty for fungal symptoms.
FANS	Anthracnose	-0.10	Absence preferred	Mild penalty for disease symptoms.
FRR	Root rot	-0.10	Absence preferred	Mild penalty for disease symptoms.
FAS	Ascochyta	-0.10	Absence preferred	Mild penalty for disease symptoms.
FRUS	Rust	-0.10	Absence preferred	Mild penalty for disease symptoms.
FANO	Angular leaf spot	-0.10	Absence preferred	Mild penalty for disease symptoms.
FALT	Alternaria	-0.10	Absence preferred	Mild penalty for disease symptoms.
FOT	Other fungi	-0.10	Absence preferred	Mild penalty for other fungal symptoms.
BV	Bacteria and virus symptoms	-0.10	Absence preferred	Mild penalty for general bacterial or viral symptoms.
BMN	Bean common mosaic necrosis virus	-0.10	Absence preferred	Mild penalty for viral symptoms.
BMV	Bean common mosaic virus	-0.10	Absence preferred	Mild penalty for viral symptoms.

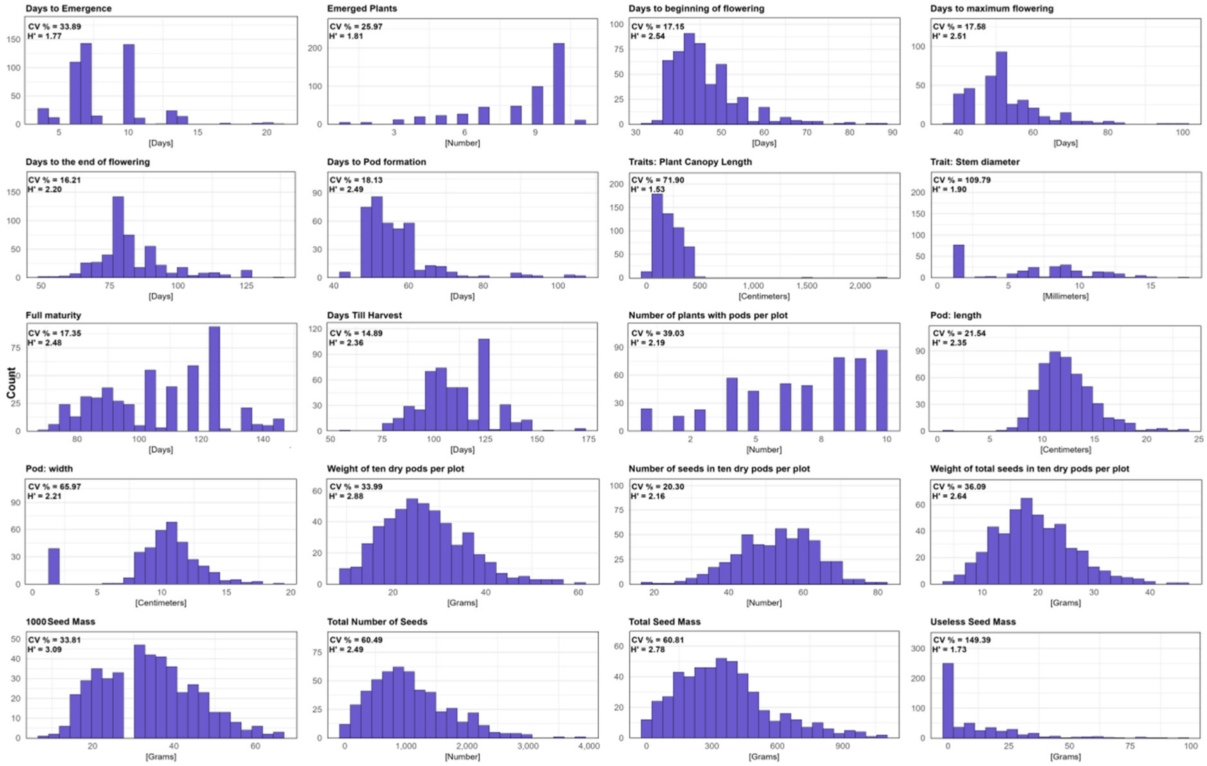

Figure S1, Frequency distributions of 20 quantitative traits measured across 507 phenotyped lines, H′ indicates the Shannon diversity index, and CV (%) indicates the coefficient of variation,

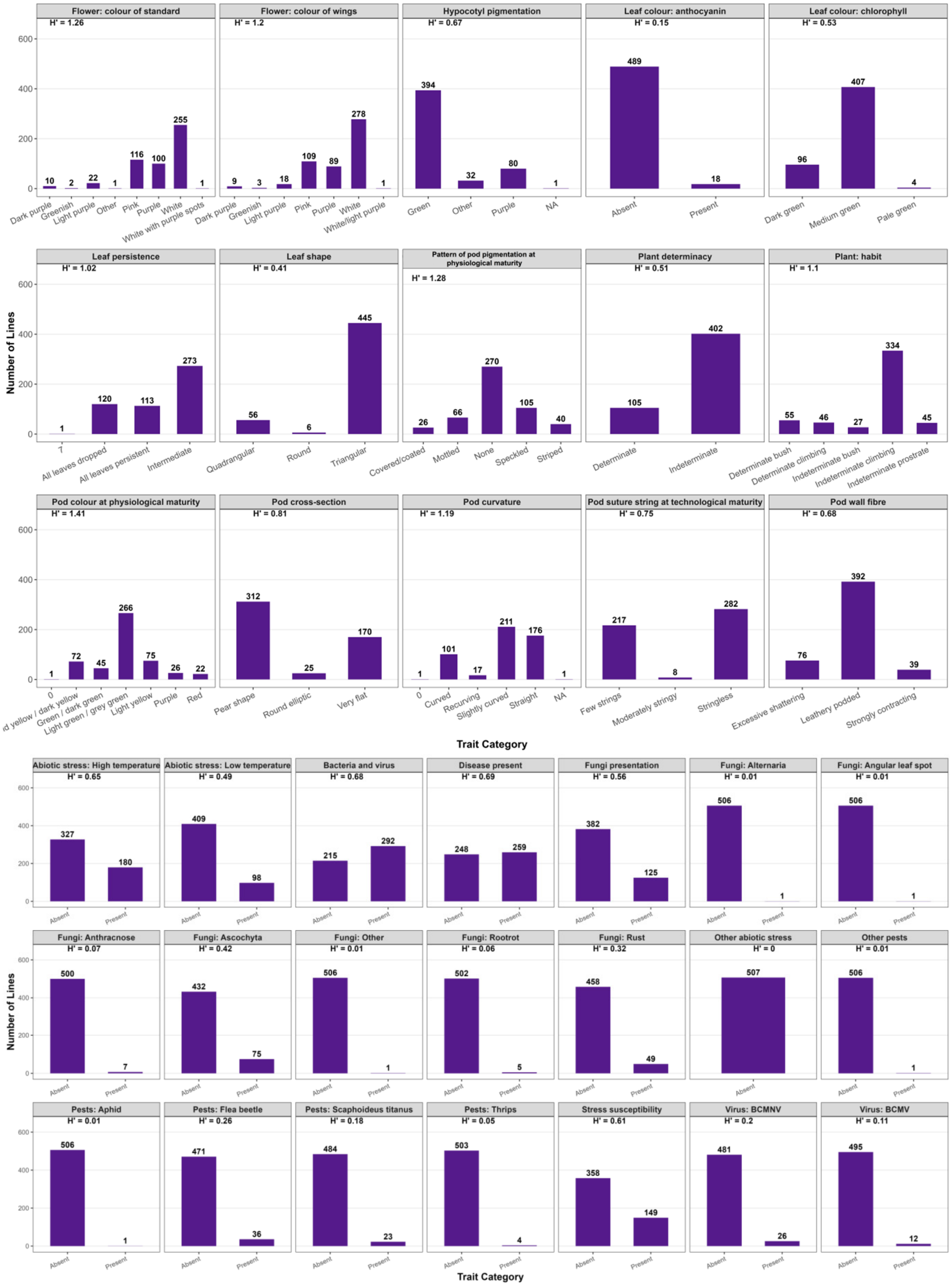

Figure S2, Frequency distribution of qualitative morphological and stress-related traits in the R-core common bean lines, The Shannon diversity index (H′) is shown for each trait class,

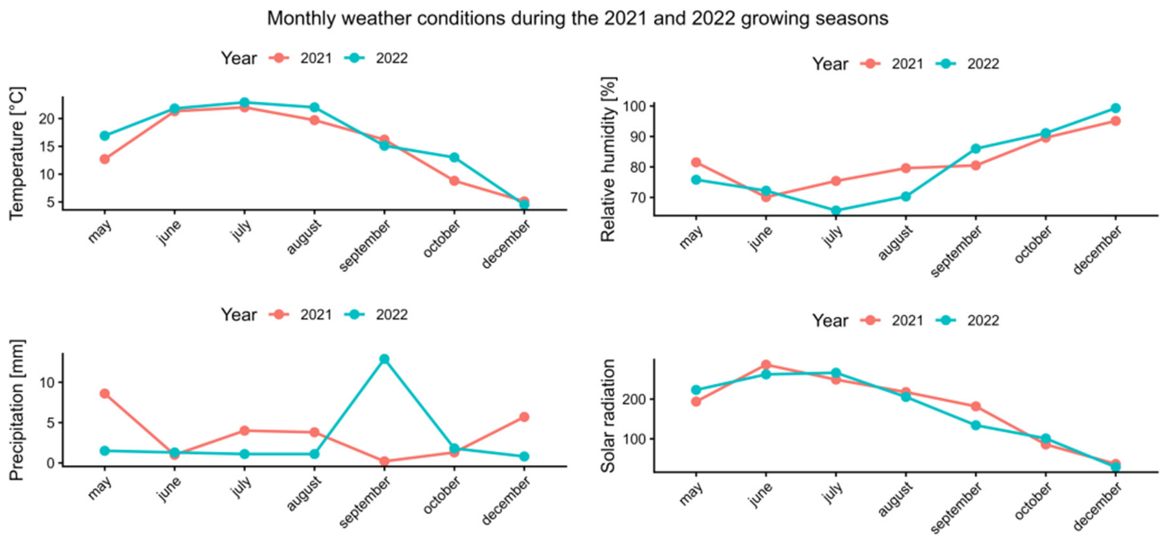

Figure S3, Monthly temperature, precipitation, relative humidity, and solar radiation during the 2021 and 2022 growing seasons,
